# Cardiomyocyte external mechanical unloading activates modifications of α-actinin differently from sarcomere-originated unloading

**DOI:** 10.1111/febs.16925

**Published:** 2023-08-17

**Authors:** Christopher Solís, Chad M. Warren, Kyle Dittloff, Elisabeth DiNello, R. John Solaro, Brenda Russell

**Affiliations:** Department of Physiology and Biophysics, Center for Cardiovascular Research, University of Illinois at Chicago, IL, USA

**Keywords:** calponin homology domain, HCM, HFrEF, myotrope, sarcopenia

## Abstract

Loss of myocardial mass in a neonatal rat cardiomyocyte culture is studied to determine whether there is a distinguishable cellular response based on the origin of mechano-signals. The approach herein compares the sarcomeric assembly and disassembly processes in heart cells by imposing mechano-signals at the interface with the extracellular matrix (extrinsic) and at the level of the myofilaments (intrinsic). Experiments compared the effects of imposed internal (inside/out) and external (outside/in) loading and unloading on modifications in neonatal rat cardiomyocytes. Unloading of the cellular substrate by myosin inhibition (1 μM mavacamten), or cessation of cyclic strain (1 Hz, 10% strain) after preconditioning, led to significant disassembly of sarcomeric α-actinin by 6 h. In myosin inhibition, this was accompanied by redistribution of intracellular poly-ubiquitin K48 to the cellular periphery relative to the poly-ubiquitin K48 reservoir at the I-band. Moreover, loading and unloading of the cellular substrate led to a three-fold increase in post-translational modifications (PTMs) when compared to the myosin-specific activation or inhibition. Specifically, phosphorylation increased with loading while ubiquitination increased with unloading, which may involve extracellular signal-regulated kinase 1/2 and focal adhesion kinase activation. The identified PTMs, including ubiquitination, acetylation, and phosphorylation, are proposed to modify internal domains in α-actinin to increase its propensity to bind F-actin. These results demonstrate a link between mechanical feedback and sarcomere protein homeostasis via PTMs of α-actinin that exemplify how cardiomyocytes exhibit differential responses to the origin of force. The implications of sarcomere regulation governed by PTMs of α-actinin are discussed with respect to cardiac atrophy and heart failure.

## Introduction

A small, weak heart is a major clinical problem. This often results from inactivity and unloading triggered by such events as prolonged bed rest or space flight. Exercise during these circumstances can mitigate some of the problems [1–3]. An enlarged, dilated cardiomyopathic heart in failure is also weak, and some mitigation with reverse remodeling can result by the use of a left ventricular assist device (LVAD) as a bridge to transplant [4–6]. Despite the clinical importance of decreasing heart size, little is known about the removal of ventricular mass after alleviating the hemodynamic load on the heart. Better mechanistic understanding might provide novel ideas for appropriate amelioration of ventricular atrophy. The goal here is to assess how myocyte size proceeds during unloading.

The body of knowledge exploring the biochemical and structural implications of myocardial unloading and sarcomere atrophy is lacking compared to studies on myocardial muscle overload and sarcomere addition. An early study by George Cooper IV’s group surgically unloaded the cat ventricular papillary muscles by severing the cordae tendineae *in vivo*, which led to substantial atrophy of the muscle mass in one week despite the myocytes remaining electrically integrated to the ventricle [7,8]. Reloading of papillary muscle restored sarcomeric protein content. The best evidence of reverse remodeling of human myocardium comes from patients after LVAD implantation where chronic hemodynamic unloading decreases left ventricular mass and myocyte size [4,9]. Heterotopic transplant of a second unloaded, and non-pumping heart into the abdomen of a rat resulted in a reduction in cardiac muscle cell size [10,11]. A recent review discusses the many unanswered questions relating to the regulation of heart cell mass in various unloaded heart cell models, specifically concerning sarcomeric protein half-life decrease and degradation increase [12]. Much has also been learned about skeletal muscle atrophy with unloading [13] or tenotomy [14,15], but heart and skeletal muscle processes may differ.

The questions posed here explore what happens with unloading and whether there is a distinguishable cellular response to unloading of forces based on the origin of the mechanical stimulus, that is, inside-out or outside-in [16]. The rationale for the selection of two model systems of diminished mechanical tension inducing atrophy by unloading where the forces in one are initialed within the sarcomere, and in the other where the forces are generated by external manipulation of the substrate on which the neonatal rat ventricular myocytes (NRVMs). are grown. First, for inside-out forces the internal sarcomeric tension is inhibited with the myosin inhibitor mavacamten (Mava), or with omecamtiv mecarbil (OM) as a comparative force generating myosin activator. Second, for outside-in, the external force is reduced in NRVMs are initially being cyclically strained to increase muscle mass above baseline. Subsequent turning off the cyclic flexing allows examination of the disassembly of sarcomeres as the NRVM mass returns to baseline. Biochemical and structural techniques are used to analyze the changes resulting from the reduction in tension of myosin or by removal of the cyclic strain applied to the cellular substrate.

This data demonstrate that internal and external unloading promote different biochemical and structural modifications in cardiac myocytes. Differences found suggest that different signaling pathways are activated inside-out versus outside-in therefore several kinases were examined. Moreover, these different responses induce different post-translational modifications (PTMs) in α-actinin associated with extracellular signal-regulated kinase (ERK) and focal adhesion kinase (FAK), as well as localized disassembly of sarcomeres via altered signaling in the ubiquitin pathway. These results have important implications for how mechanical feedback arising from diverse myocardial stresses can result in diverse responses.

## Results

**Fig. 1.**
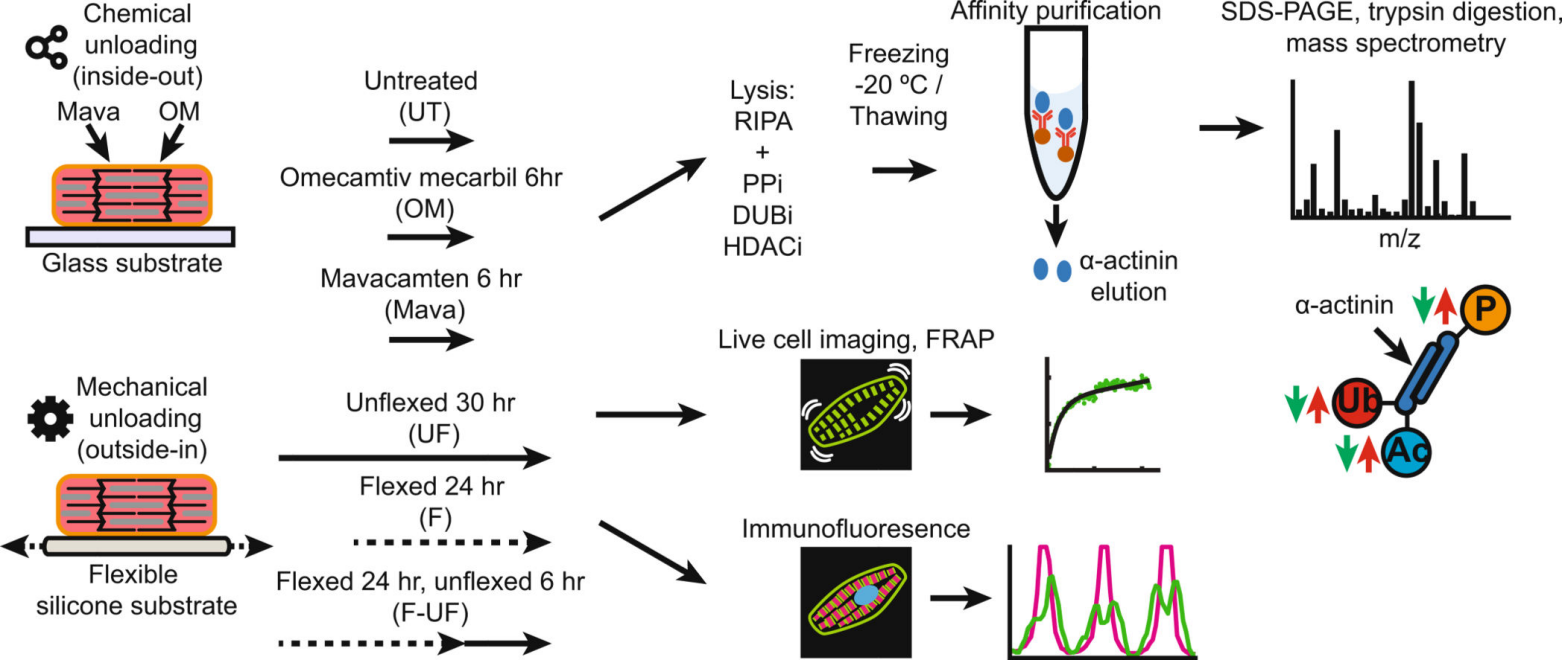
Experimental Approach to study the response of cardiac muscle cells to inside-out and outside-in forces. Two experimental paradigms of mechanical force stimulation were tested to study the effects of mechanical forces on cardiac sarcomere assembly and disassembly and the associated chemical and biophysical changes. The first approach was an intrinsic stimulus elicited by drugs that modify myosin contractility (i.e. inside-out). The second approach was an extrinsic stimulus from varying strain applied to the cardiomyocyte attachment substrate (i.e. outside-in). The inside-out approach consisted of treating NRVMs with the myosin inhibitor Mavacamten (1 μM, 6 h) or the myosin activator Omecamtiv Mecarbil (0.5 μM, 6 h). The outside-in approach consisted of 1 Hz cyclic strains for 24 h (loaded) followed by 6 h rest (unloaded) of cultured neonatal rat ventricular myocytes (NRVMs) and compared with continuously strained cultures. These two approaches were compared by quantitative immunofluorescence, targeted proteomics of α-actinin, and fluorescence recovery after photobleaching experiments (FRAP).

**Fig. 2.**
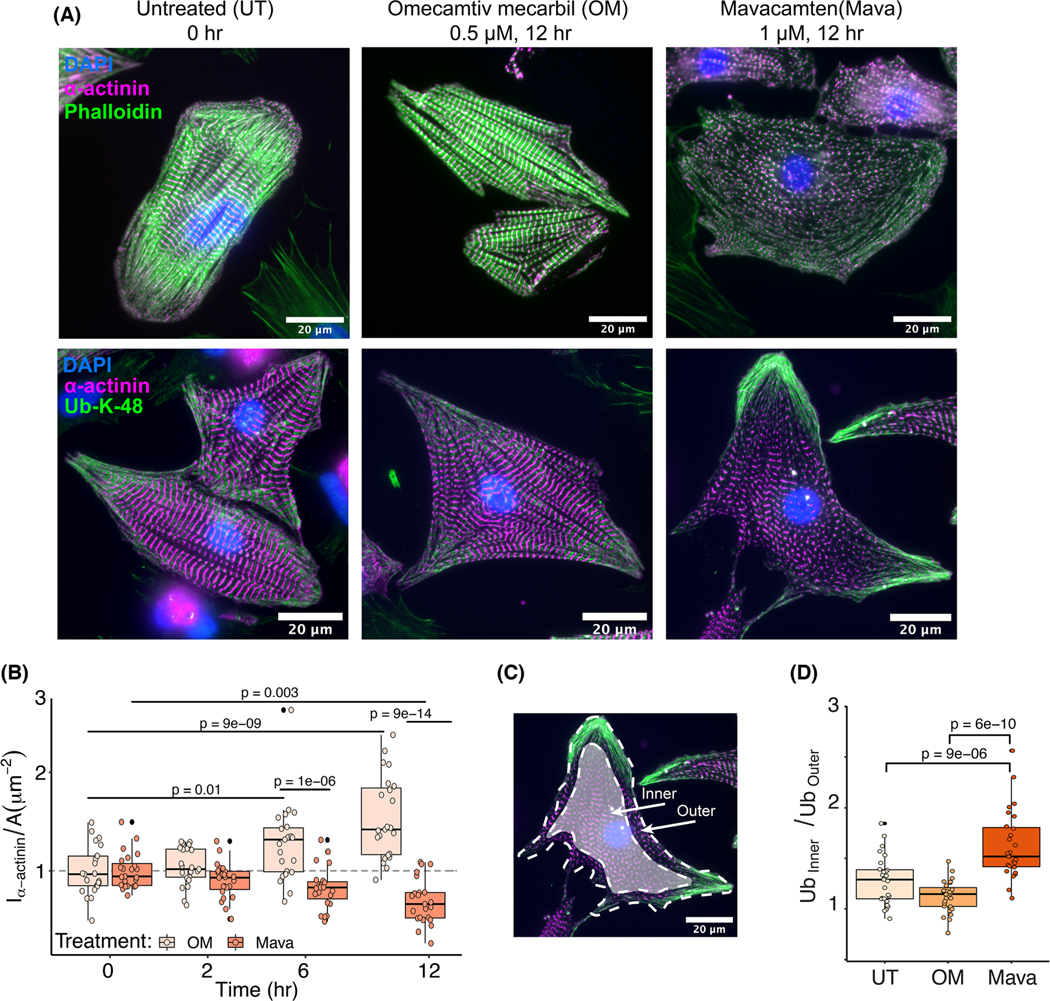
Sarcomere disassembly begins at the outer annulus of the cell with diminished myosin tension. (A) Untreated (UT) NRVMs were compared with NRVMs treated with OM (0.5 M) or Mava (1 μM) at 12 h after fixing and staining with α-actinin (magenta) and phalloidinrhodamine (top panels) or α-actinin (magenta) and oligo-Ub-K48 (green) antibodies (bottom panels) (scale, 20 μm). (B) Sarcomeric α-actinin content per cell area was quantified for NRVMs treated with OM and Mava over the course of 12 h. (C) The distribution of oligo-K48 ubiquitin between the non-striated annulus 5 μm from the cell edge (outer) and central sarcomeric region (inner) was quantified. (D) The oligo-Ub-K48 content of the outer relative to middle between UT, OM, and Mava (error bars indicate mean ± standard deviation; 27 measurements from independent cells per treatment, or nine quantified cells from each *N* = 3 independent cell culture; multicomparison tests use Tukey’s honest significance test).

The functional consequences of unloading cardiac muscle cells were defined between two experimental paradigms (Fig. 1). Treating NRVMs with Mava for 12 h led to the disassembly of sarcomeres, while no visual differences were seen when comparing OM to UT (Fig. 2A, top panels). Quantification of α-actinin content per cell area showed that Mava drove significant disassembly at 6 h compared to OM, and the protein loss was significant by 12 h when compared to baseline conditions at 0 h. Particularly, the alterations in sarcomeres occurred at the cell periphery. Conversely, OM increased α-actinin content significantly by 6 h, and this gain further increased by 12 h. This 6 h threshold of significant differences is used in subsequent experiments. Taken together, the disassembly of sarcomeres occurred with the reduction of myosin tension, which was initiated at the cell periphery, on a scale of hours.

Protein homeostasis was studied by assessing the subcellular distribution of the oligo-ubiquitin K48 motif as a function of the myosin inhibitors or activators. The oligo-ubiquitin K48 modification is responsible for tagging proteins committed for proteasomal degradation [17]. As such, immunostains with an antibody sensitive to this modification interrogate the proteostatic environment within the cardiac muscle cells. Fig. 2A (bottom panels, *green*) shows a redistribution of the oligo-ubiquitin K48 proteoforms to the outer 5 μm annulus with Mava when compared to UT and OM. This was quantitatively established in Fig. 2B–D. These data suggest that diminished sarcomeric tension from myosin inhibition redistributes the oligo-ubiquitin K48 proteoforms to the cell periphery.

**Fig. 3.**
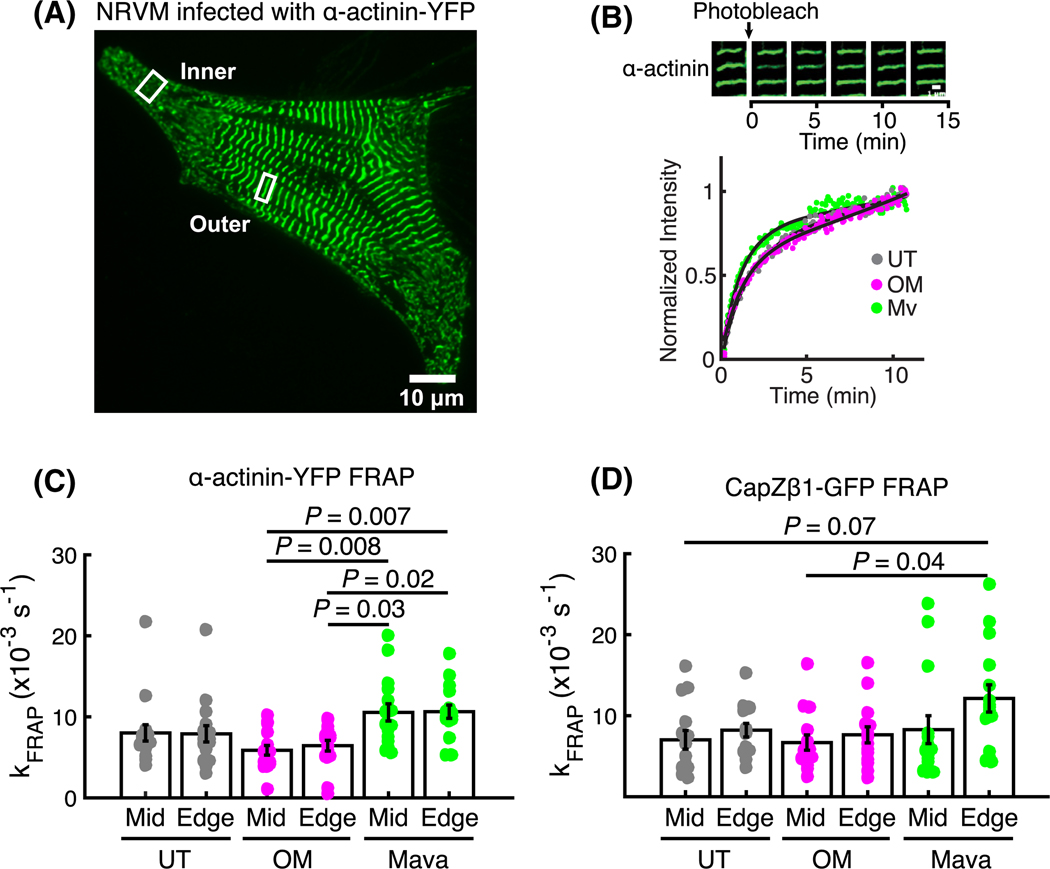
Diminished myosin tension increases α-actinin and CapZ exchange dynamics. The dynamic exchange of the Z-disc proteins α-actinin and CapZ in response to chemical unloading were probed in NRVMs infected with adenoviral constructs expressing α-actinin-yellow fluorescent protein (YFP) or CapZβ1-GFP. (A) The sarcomeric (inner) and non-sarcomeric (outer) fluorescent protein pools were photobleached as in (B) while tracking the time-dependent recovery of fluorescence from dynamic exchange of proteins at the Z-discs (12 min imaging per region of interest). The FRAP recovery kinetics (k_FRAP_) are summarized for α-actinin-YFP (C) and CapZβ1-GFP (D) in response to treatments with OM and Mava for 6 h (error bars indicate mean ± standard deviation, 27 measurements from independent cells per treatment, or nine quantified cells from each *N* = 3 independent cell culture; multicomparison tests use Tukey’s honest significance test).

The functional basis underlying myofibrillar disassembly was assessed by measuring the dynamics of exchange of the sarcomere protein α-actinin in response to 6 h treatments with OM or Mava. NRVMs expressing α-actinin-yellow fluorescent protein (YFP) were subjected to a fluorescence recovery after photobleaching (FRAP) experiment to interrogate the dynamics of protein exchange at the outer 5 μm annulus (outer) and the sarcomeres with Z-discs (inner), Fig. 3A. The typical fluorescence recovery traces after photobleaching single sarcomeres, Fig. 3B, showed different characteristic recovery rates for each treatment, which were quantified in Fig. 3C. As the k_FRAP_ is proportional to the dynamics of exchange, this parameter informs that α-actinin-YFP exchange dynamics are increased with Mava at both the - pre-myofibrils and mature Z-discs. To understand whether this is a generalized mechanism that applies to other sarcomeric proteins, NRVMs were also infected with CapZ-green fluorescent protein (GFP) (Fig. 3D). These cells demonstrated increased dynamics of exchange only at the non-striated outer region. In support of this finding, studies in a mutant α-actinin showed slower FRAP dynamics and increased binding affinity for F-actin relative to wild-type [18]. Conversely, other proteins like F-actin show increased FRAP dynamics with OM [19]. Together, these data indicate that diminished tension caused by the myosin inhibitor increased the dynamics of exchange of Z-disc proteins like α-actinin and CapZ.

**Fig. 4.**
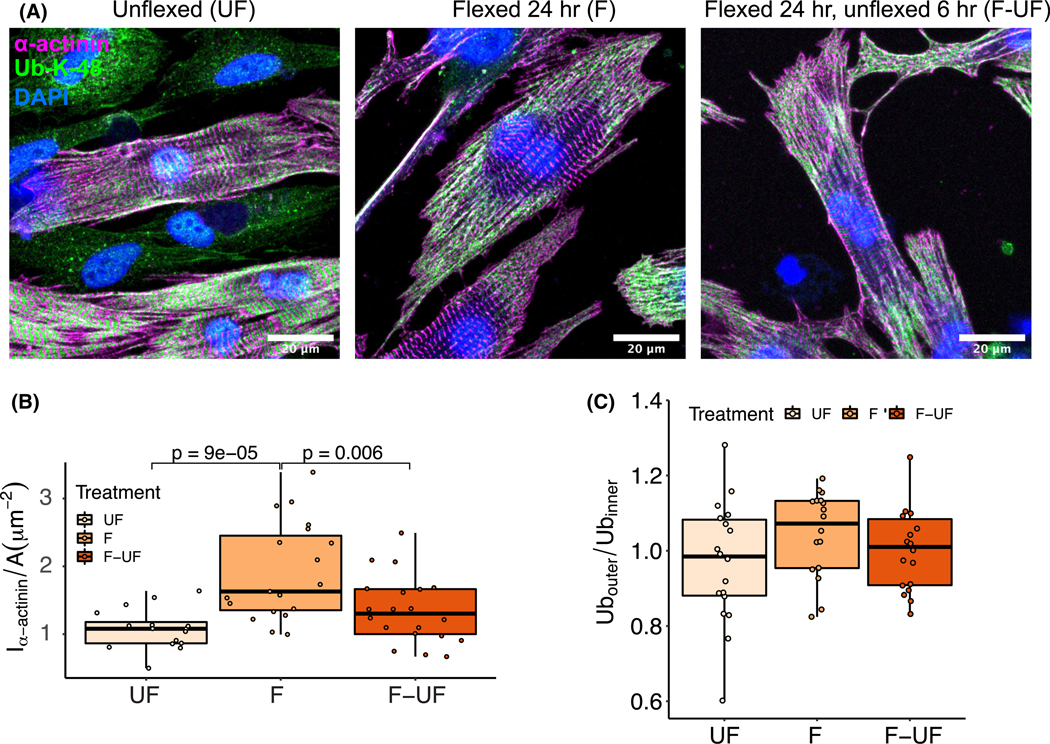
Sarcomere assembly due to external loading is reversed by unloading. (A) Unflexed (UF) NRVMs are compared with NRVMs treated with 24 h flexing (F), or 24 h flexing followed by 6 h rest (F-UF) after fixing and staining with α-actinin and oligo-Ub-K48 antibodies (scale, 20 μm). (B) α-actinin content per cell area shows significant gain in sarcomeric α-actinin after F (*P* < 0.001) and significant loss of sarcomeric α-actinin after the F-UF treatment (*P* < 0.01). (C) The proportion of the oligo-Ub-K48 content in the outer 5 μm annulus is not significantly different between UF, F, and F-UF outer relative to inner regions (error bars indicate mean ± standard deviation; 27 measurements from independent cells per treatment, or nine quantified cells from each *N* = 3 independent cell culture; multicomparison tests use Tukey’s honest significance test).

The second paradigm of regulation of tension is for externally unloading NRVMs by reducing the cellular substrate tension in cyclically strained NRVM cell cultures. This is done by culturing NRVMs in 6-well plates with flexible membranes that are cyclically strained by applying vacuum pressure from below, leading to a 10% lengthening of the substrate at a frequency of 1 Hz. Unflexed wells (UF) were compared to 24 h flexed (F) or 24 h flexed followed by a 6 h period of rest (F-UF). Myocytes fixed and stained after each treatment showed no visual differences in α-actinin or oligo-ubiquitin K48 motif distribution (Fig. 4A). However, α-actinin content increased with F relative to UF, while F-UF significantly decreased relative to F and was undistinguishable from UF, suggesting that the 6 h period of rest was sufficient to regress the levels of sarcomere content back to baseline (Fig. 4B). Quantifying the oligo-ubiquitin K48 (Fig. 4C) showed no redistribution to the outer annulus in contrast with Fig. 2D. Taken together, mechanical loading from cellular substrate cyclical deformation increases sarcomere assembly, while acute cessation of cyclical strain is sufficient to decrease sarcomere content back to baseline without altering oligo-ubiquitin K48 motif distribution.

**Fig. 5.**
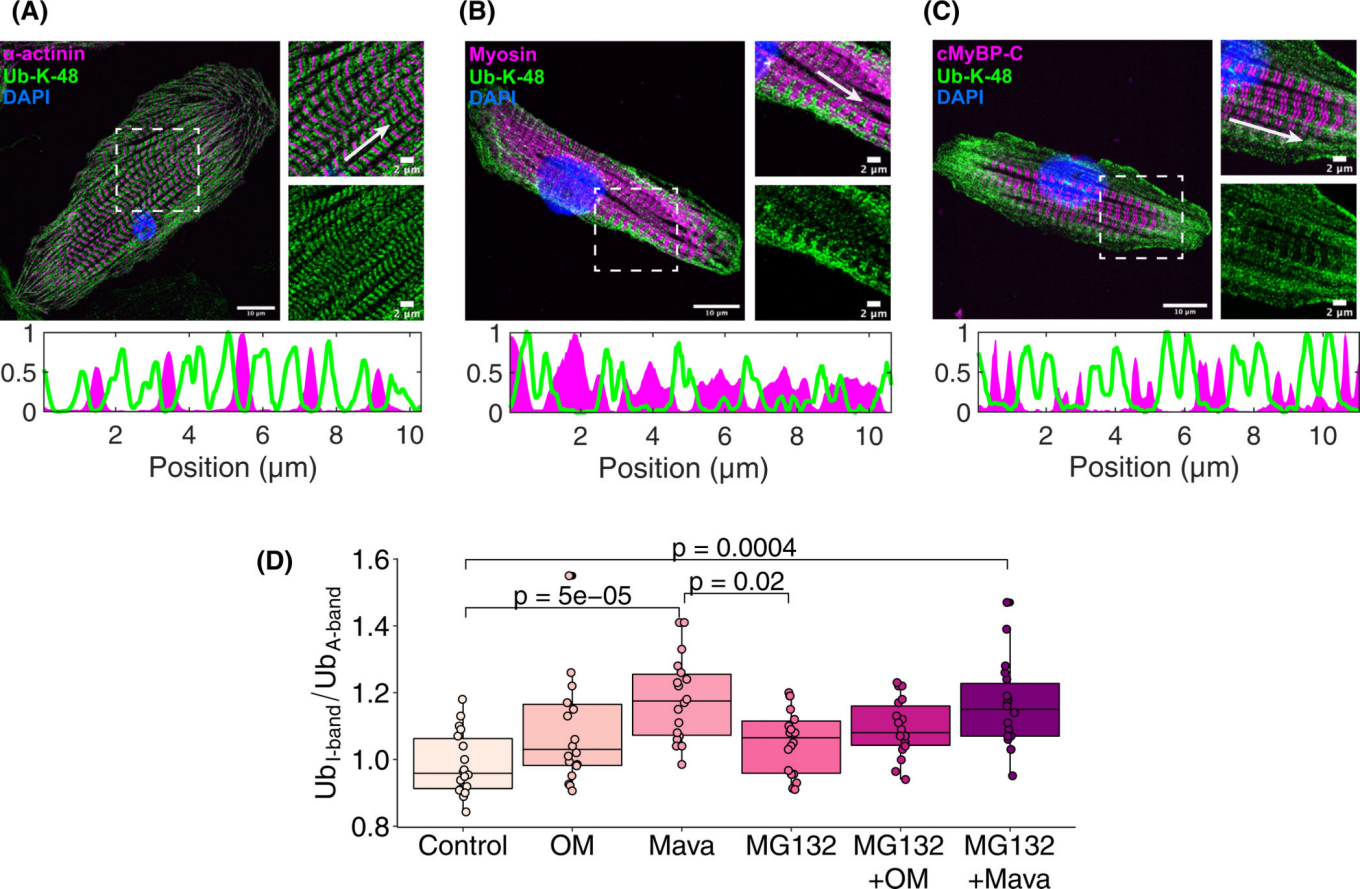
Sarcomeric oligo-K48-linked ubiquitin localizes at the I-band and increases with unloading from myosin inhibition. Super-resolution fluorescence immunostains depict oligo-Ub-K48 distribution (green) relative to (A) α-actinin, (B) myosin, and (C) cMyBP-C (magenta). The dashed squares indicate the location of the magnified images on the right (Scale bar of enlarged and magnified images is 10 and 2 μm respectively). White arrows 10 μm long show direction and fluorescence intensity profile of ubiquitin-K48 (green) and the selected sarcomeric proteins (magenta). (D) Quantification of the I-band oligo-Ub-K48 relative to the A-band oligo-Ub-K48 fixed at 6 h after treatment (error bars indicate mean ± standard deviation; 27 measurements from independent cells per treatment, or 9 quantified cells from each *N* = 3 independent cell culture; multicomparison tests use Tukey’s honest significance test).

Next, super-resolution imaging was applied to assess the redistribution oligo-ubiquitin K48 modification with internal unloading by Mava. The sarcomeric location of the ubiquitin K48 motif was limited to the I-band as its signal did not colocalize with α-actinin, myosin heavy chain, or myosin binding protein C (Fig. 5A–C). Quantifying the relative abundance of ubiquitin K48 motif at the I-band relative to the A-band showed enrichment in ubiquitin K48 with Mava relative to controls. A similar distribution of ubiquitin K48, including the peripheral enrichment, was seen at the I-band of human-induced pluripotent stem cell cardiomyocytes (Fig. S1). Furthermore, applying the proteasome inhibitor MG132 along with the drugs shows that the ubiquitin K48 motif was further enriched by the net loss of differences between MG132 and MG132 + Mava treatments (Fig. 5D). The poly-UbK48 antibody was further validated by increasing ubiquitin K48 motif abundance in cell lysates with exposure to MG132 (Fig. S2). This indicates that the sub-sarcomeric location of the ubiquitin K48 motif was limited to the I-band and its enrichment in ubiquitin K48 motif occurred with diminished myosin cell tension.

**Fig. 6.**
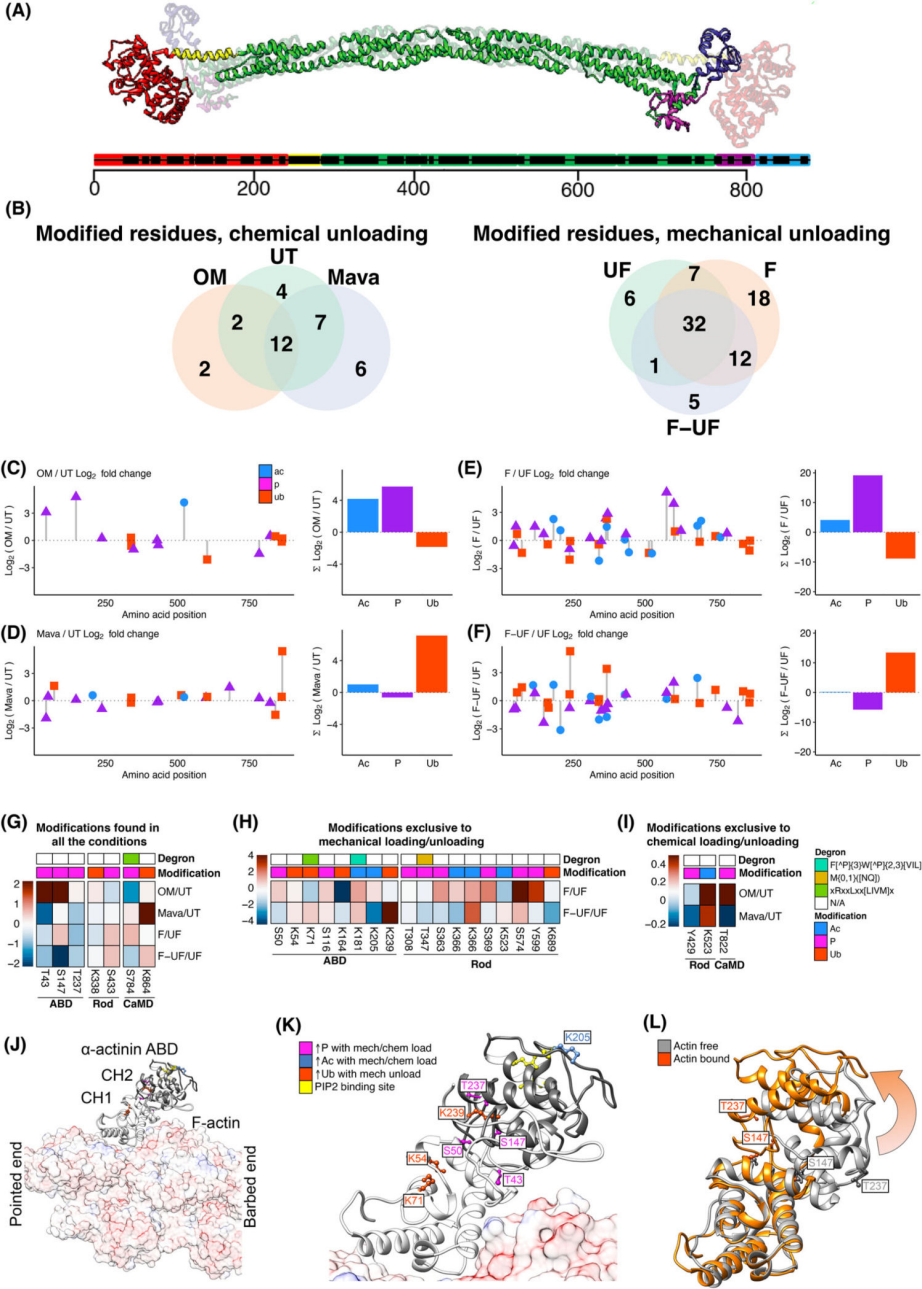
Post-translational content of α-actinin reveals alterations of actin-binding interface and protein degradation sites. (A) Crystal structure of α-actinin-2 (PDB: 4D1E) Displaying color-coded domains and their arrangement across the full sequence (ABD, red; neck region, yellow; spectrin-like repeats or rod regions, green; CaMDs composed of EF-hand motifs, magenta and purple). (B) The mechanical intervention (*outside-in*: F, F-UF) causes more post-translational modification changes compared with the chemical intervention (*inside-out*: Mava, OM). (C–F) Log_2_ fold changes in acetylation, ubiquitination, and phosphorylation are displayed across the rat α-actinin-2 linear sequence (NCBI ref. seq.: NP_001163796.1). (C) OM and (D) Mava Log_2_ fold change relative to UT are presented across the linear sequence and summarized in frequency histograms. (E) F and (F) F-UF a Log_2_ fold change relative to UF are presented across the linear sequence and summarized in frequency histograms. Heatmap analysis of (G) modified residues found in all the treatments, (H) modified residues only found in the *outside-*in dataset, and (I) residues only found in the *inside-out* dataset. Top lines show modification of each residue and predicted degron sites. (J) Homology model of the actin binding domain (ABD) of α-actinin-2 (template, PDB ID 7R94) illustrates the spatial arrangement of CH1 and CH2 domains relative to actin, and (K) a close view depicting modified residues from G–I. (L) ABD models of actin-free (template, PDB ID 4D1E) and actin-bound (template, PDB ID 7R94) α-actinin-2 show relative displacement of CH1 and CH2 domains by arrow and spatial rearrangements of S237. Models were prepared for illustration in UCSF Chimera (version 1.6, build 42360).

To understand how outside-in mechano-signaling pathways versus inside-out myosin tension control converge on sarcomeric proteins to modulate assembly and disassembly, targeted mass spectrometry was employed to interrogate the post-translational composition of α-actinin as shown in the structure depicted in Fig. 6A. This revealed that the external loading led to more PTM changes than the internal, chemical approach (Fig. 6B). The modifications studied included acetylation, phosphorylation, and ubiquitination. Displaying the log_2_-fold changes in PTMs at the single-residue level resolved quantitative changes in the post-translational content responsive to both internal or external loading or unloading (Fig. 6C–F, Fig. S3). First, a relative enrichment in all the PTMs was found at the N and C terminus of α-actinin and away from the structurally stable spectrin-like domains. This could be due to a larger number of residues being buried inside the structurally stable rod regions composed of spectrin-like domains, particularly when considering that the dimeric state has the most overlap at that region [20]. Second, when grouping the log_2_-fold changes for each PTM, acetylation increased across all treatments or remained unchanged. Changes became more distinctive with phosphorylation increasing with OM and flexed but decreasing with Mava and flexed-unflexed (F-UF). Conversely, ubiquitination decreased with OM and flexed, and increased with Mava and F-UF (Fig. 6C–F). Taken together, these results show that the external approach to manipulate loading and unloading activates more PTMs relative to the internal approach. In these circumstances, loading drives upregulation of phosphorylation and downregulation of ubiquitination, while unloading causes downregulation of phosphorylation and upregulation of ubiquitination.

To further categorize these modifications at the single-residue level, the relative abundance of each modified residue was directly compared across all possible logical relations defined by the experimental treatments. Hence, modified residues were grouped in heatmaps by residue number in the first dimension (*x*-axis) and by treatment (*y*-axis) in the second dimension (a) being found across all the treatments (Fig. 6G), (b) only in the mechanical approach (Fig. 6H), or (c) only in the chemical approach (Fig. 6I). Overall, the mechanical approach exhibited more unique changes in PTMs, with the vast majority concentrating at the actin-binding domain when accounting for the amino acid length of each region. Furthermore, selected residues are listed based on their differential response to treatments. At the actin binding domain (ABD, aa 1–260), S43 and T237 phosphorylation increased with OM/UT relative to Mava/UT, S50 phosphorylation increased with F/UF relative to F-UF/UF, S147 phosphorylation increased with OM/UT and F/UF relative to Mava/UT and F-UF/UF, K205 acetylation increased with F/UF relative to F-UF/UF, and K71 and K239 acetylation increased with OM/UT and F-UF/UF relative to Mava/UT and F-UF/UF. At the rod region (aa 261–750), S363, S369, and Y599 phosphorylation increased with F/UF relative to F-UF/UF; K366 acetylation increased with F/UF relative to F-UF/UF; and K864 ubiquitination increased with Mava/UT and F-UF/UF. At the calmodulin-like domain (CaMD, aa 751–892), S784 phosphorylation increased with F/UF relative to F-UF/UF, K864 ubiquitination increased with Mava/UT and F-UF/UF relative to OM/UT and with F-UF/UF, and T822 phosphorylation increased with OM/UT relative to Mava/UT. Some of these stimulus-responsive modifications are part of predicted degron sites such as K71 in the ABD and S784 in the CaMD [21].

Because the ABD is the region most enriched in PTMs per amino acid, several sites were mapped into structural models of α-actinin-2. Using a crystal structure of T-plastin bound to F-actin as a template (PDB ID: 7R94) [22], a homology model of the α-actinin-2 binding domain was built based on its amino acid sequence (NCBI ref. seq.: NP_001163796.1). A second homology model of α-actinin-2 bound to actin (Fig. 6J) shows the CH1 domain to be in direct contact with the actin surface while the CH2 shows no interactions with actin, in agreement with other structural and biochemical work that highlights the CH1 domain as responsible for providing actin binding while CH2 is unnecessary for this task [23]. When zooming into this structure (Fig. 6K), it is possible to classify these residues as phosphorylation sites that increase with mechanical load (S50), phosphorylation sites that increase with the chemical approach (T237, T43), phosphorylation sites that increase with chemical and mechanical load (S147), and acetylation sites that increase with mechanical load (K205). Prior residues proposed to be involved in phosphatidylinositol 4,5-bisphosphate (PIP2) interactions are also highlighted (K169, K192, and R163) [20]. Except for the putative PIP2-interacting residues, this representation finds all these modified residues positioned at sites involved in CH1 to CH2 domain interactions rather than in sites responsible for actin binding. On the other hand, ubiquitination in this region was only found in residues K54, K71, and K239 undergoing mechanical unloading. Lastly, the homology model of the actin-bound α-actinin-2 binding domain (orange) and actin-free states (gray) highlight the relative structural displacement with the S237 phosphorylation site exhibiting the highest displacement (Fig. 6L). Together, these data provide the annotation of stimuli-responsive PTMs along with their structural location that may be relevant to control of protein dynamics and homeostasis.

**Fig. 7.**
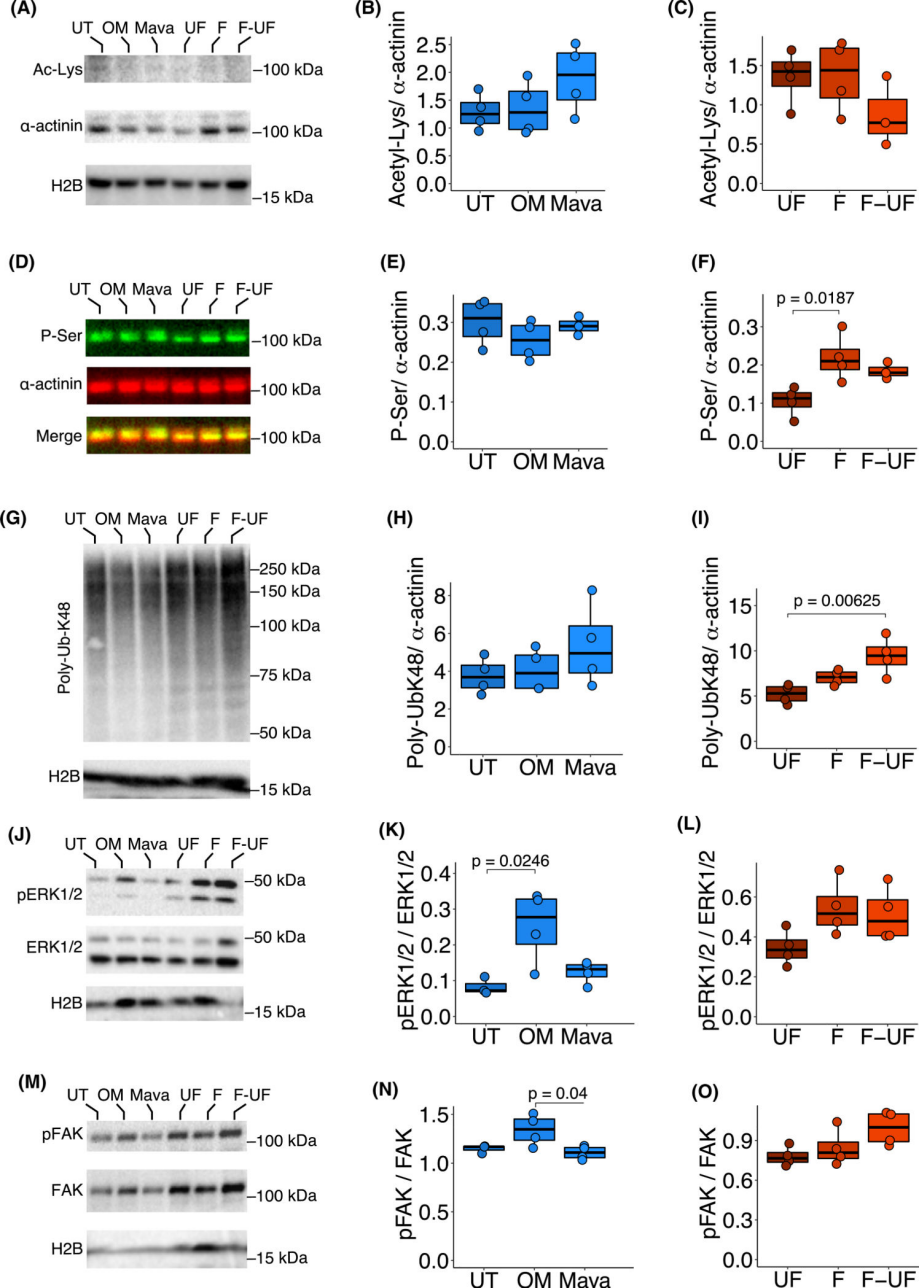
Phosphorylation, ubiquitination, ERK, and focal adhesion kinase (FAK) activity are differentially sensitive to internal and external loading and unloading. (A) Representative Acetyl-lysine and α-actinin western blots of NRVM cell lysates under untreated (UT), OM, Mava, unflexed (UF), flexed (F), and flexed-unflexed (F-UF) conditions. (B, C) Quantification of the acetyl-lysine content relative to α-actinin. (D) Representative phosphoserine western blot overlays with α-actinin for the same samples as in (A). (E, F) Quantification of the phosphoserine content relative to α-actinin. (G) Representative oligo-Ub-K48 and α-actinin western blots for the same samples as in (A). (H, I) Quantification of the oligo-Ub-K48 content relative to α-actinin. (J) Representative pERK1/2, total ERK1/2 western blots for the same samples as in (A). (K, L) Quantification of the pERK1/2 content relative to total ERK1/2. (M) Representative pFAK, and total FAK western blots for the same samples as in (A). (N, O) Quantification of pFAK relative to the total FAK. H2B is used as a loading control. *N* = 4 independent cell cultures for all the treatments; multicomparison tests use Tukey’s honest significance test.

To define how these specific modifications change across the cellular proteome, western blot analysis was done on whole cell lysates. Specifically, the acetyl lysine content relative to α-actinin was not significantly different across all the tested conditions (Fig. 7A–C, Fig. S4). However, serine phosphorylation showed a co-distribution with the α-actinin band, and again in the phospho-serine modification that significantly increased in F relative to UF (Fig. 7D–F, Fig. S5). This was similar to observations in Fig. 6C,E, showing phosphorylation to increase under increasing loads in both chemical and mechanical experiments. The mass spectrometry analysis revealed gains in phospho-Thr modifications with increasing load, but such modifications were not confirmed by the western blot (Figs 7 and S6). Lastly, Ub-K48 signal increases with F-UF relative to UF, which is also recapitulated by a net increase in ubiquitination (Fig. 7G–I, Fig. S7). Taken together these results showed that cyclic mechanical flexing elicits serine-specific phosphorylation in response to cyclic mechanical strain and Ub-K48 accumulation with mechanical unloading post-cyclic mechanical strain.

Prediction of phosphorylation sites in α-actinin-2 using an artificial neural network method [24] revealed phosphorylation sites and predicted kinases for the phosphorylated residues identified by mass spectrometry in this study (Table S1). To interrogate specific signaling pathways that may be involved in such PTMs, western blot analyses of selected kinases were conducted. The phospho-extracellular signal-regulated kinase 1/2 (ERK1/2) signal relative to total ERK1/2 changed with OM relative to UT, but not in any of the mechanical stimulation treatments (Fig. 7J–L). Phospho-FAK relative to total FAK was significantly higher with OM relative to UT (Fig. 7M–O). Changes in phospho-protein kinase A (PKA) to total PKA and phospho-protein kinase C (PKC) epsilon to total PKA signals were not detected in all the tested conditions (Figs S8–S11). Taken together, these data show that activation of specific signals depended on the modality of force being applied.

## Discussion

Internal loading and unloading in NRVMs in culture via myosin inhibitors is distinct morphologically and biochemically from external loading and unloading via deformation of the cellular substrate. This has implications for how myocardial mechanics may affect the development of hypertrophy in diseases like hypertrophic cardiomyopathy (HCM) and myocardial atrophy and weakening in long-term bed rest patients. The data presented led to a proposed model of how sarcomere assembly is stabilized by mechanical tension and PTM of α-actinin. With reduced internal tension there was substantial sarcomere disassembly at the outer annulus of the cell, which was associated with increased α-actinin and CapZ exchange dynamics at Z-discs. Conversely, when the cellular substrate was unloaded, sarcomere disassembly returned α-actinin content back to baseline conditions without notable changes in oligo-K48 distribution. Nonetheless, oligo-K48-linked ubiquitin not only localizes at the I-band and increases with unloading from myosin inhibition, but it also localizes to the outer cell annulus matching the regions where sarcomere loss is seen. Proteomic analysis of α-actinin indicates enrichment in phosphorylation and acetylation at the ABD could stabilize a high-affinity state of α-actinin for F-actin while unloading increases ubiquitin modification. ERK and FAK phosphorylation are two of the signaling pathways that become responsive upon cardiomyocyte loading.

The NRVMs in two-dimensional culture are fundamentally different from the adult myocytes in the whole heart, in terms of protein isoforms and sarcomeric and cytoskeletal arrangement. However, focus here is on sarcomeric α-actinin (ACTN2, ACTN3), which do not differ in relative abundance or isoforms from neonatal (P1) to young (P23) mice [25]. Although the CapZA and CapZB isoforms are expressed in neonatal and young states their relative abundance does change with age [25]. As such, α-actinin and CapZ results in this neonatal cardiomyocyte culture will mostly be relevant to the adult rodent. In this work, NRVMs are cultured at a high cell density where they beat spontaneously and form a syncytium with connexons as demonstrated by the free diffusion of calcein AM from one cell to another [26]. The myriad of differences between 2D cultured NRVMs and the adult heart denote that the biophysical models here are not necessarily applicable to the adult cells within the cardiac tissue in terms of physiologic and pathologic processes.

Newton’s Third Law states that for every action (force) there is an equal and opposite reaction. However, there is asymmetry in the structure of a striated muscle fiber which might introduce differential effects depending on the site of initiation of the force [27–29]. Thus, a biological response to mechanical forces might well depend on their direction and the orientation of the mechanotransducing proteins. In the case of loading, the mechanical force deforms proteins in the extracellular matrix, the focal adhesions, the numerous interior mechanosensors, and activation of signaling mediators including ERK1/2, FAK, and PKCƐ [28,30–32]. Instead of NRVMs, hiPS-CMs [19,33] would provide better relevance to the human than use of rodent cells. There are many 3D models that are superior to 2D in terms of cell structure and function. These include repopulating decellularized myocardial tissues [34,35], myocardial slices [36,37], and engineering myocardial tissues [38]. Importantly, cardiomyocyte growth is almost entirely driven by increased mechanical feedback that triggers the cellular growth program [39]. Nevertheless, note that almost all the research published to date with these bioengineered models study hypertrophy in loading but research into unloading is uncommon.

Detailed biochemical assessment focused on α-actinin-2, an actin bundling protein, as an index for sarcomere assembly or disassembly due to its tight interconnectivity to several sarcomere proteins [40]. α-actinin is an actin cross-linker located in the sarcomere at the Z-discs (isoforms 2 and 3) and cellular cortex (isoforms 1 and 4) [31,41]. Single point mutations at the actin-binding domain of α-actinin-4 increase its binding affinity for actin and reduce cell motility and cytoplasm dynamics, but interestingly cells are capable of generating larger forces due to a stiffer cytoskeleton that better coordinates myosin contraction [18,42]. Furthermore, α-actinin-2 was used here as a sentinel probe for activation of signaling pathways converging onto structural proteins and altering their PTM states. α-actinin was previously reported to become ubiquitinated and deacetylated upon hindlimb cast immobilization of rats [43]. In skeletal muscles, atrophy is characterized by myotube size attrition and ubiquitination of sarcomeric proteins by ubiquitin ligases such as MuRF1 and MAFbx for proteasomal degradation [44]. The central role of α-actinin as a stabilizer of sarcomeres is defined by its early incorporation into pre-myofibrillar structures called Z-bodies, from where condensation of other proteins lead to the formation of sarcomeres and provides the basis for sarcomeric α-actinin as an indicator of myofibrillar assembly [40,45]. The activation of ERK1/2 and FAK in response to increased myosin tension is noteworthy considering that activation of both signals has been implicated in cardiomyocyte hypertrophy. ERK1/2 has been used as reliable readout of hypertrophic stimulus [46]. For example, activation of ERK1/2 via Mek1 upregulation led to concentric growth of hearts and increased cardiomyocyte thickening [47]. Likewise, FAK activation is observed in response to transverse stretching of cardiomyocytes as well as due to acute pressure overload of rat hearts [28,48].

**Fig. 8.**
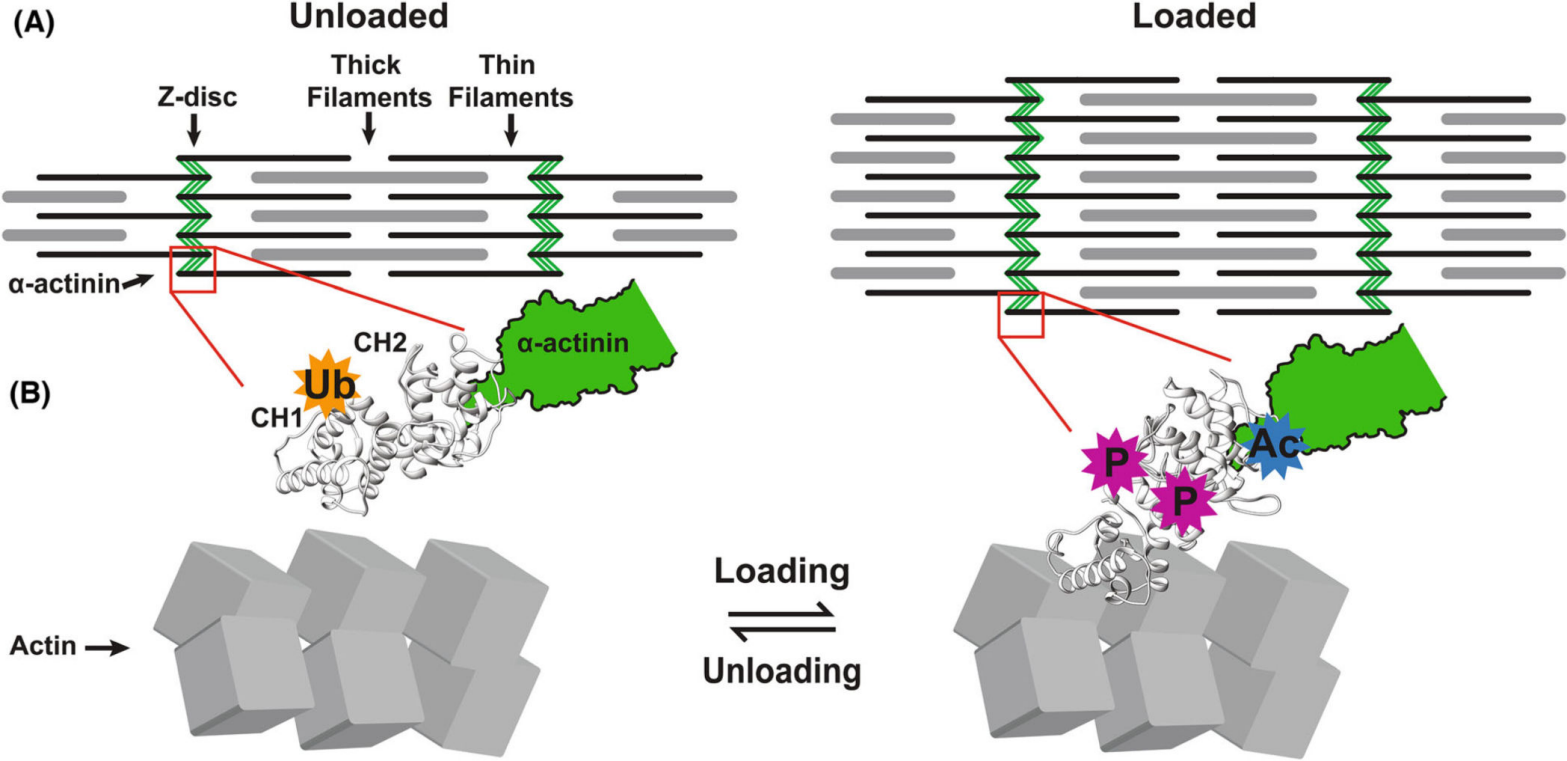
Control of sarcomere assembly via regulation of α-actinin stabilization of sarcomeres in response to loading and unloading. (A) Sarcomere growth is supported by the increased cross-linking activity of structural proteins like α-actinin-2 that bundle the thin filaments forming the Z-discs, shown in an unloaded (left) or loaded sarcomere (right). (B) α-actinin-2 is shown at the molecular scale as loosely bound to actin (left). Tight binding is promoted by increasing loading through both mechanical flexing or myosin activators with similar PTMs phosphorylation (P) and acetylation (Ac). Signaling pathways are proposed to modify α-actinin at the interphase between the CH1 and CH2 domains to increase CH1 availability to bind F-actin. However, the ubiquitination (Ub) is only greater with recovery after flexing; it is not seen with myosin inhibition at the level of the sarcomere.

Juxtaposing the load-sensitive phosphorylation sites on the α-actinin, the ABD structure allows important mechanistic conclusions to be proposed (Fig. 8). First, the phosphorylation residues that become enriched with increasing chemical (aa 43, 237), mechanical (aa 50), or both (aa 145) loads are concentrated at the interface between the CH1 and CH2 domains. Previous work has demonstrated that opening of the CH1 and CH2 domains is a necessary step to trigger binding of the α-actinin to actin [49,50]. In particular, this CH1-CH2 opening and closing transition has been characterized as an induced-fit model that is in equilibrium between the two states [50]. One hypothesis is that the negative electrostatic charge of the phosphorylation sites favors the open state of the CH1 and CH2 domains to stabilize α-actinin cross-linking of F-actin. This is also supported by the K255E single point mutation seen in the α-actinin-4 isoform (K239 in rat α-actinin-2) that destabilizes the CH1-CH2 interface by substitution of a positive charge for a negatively charged amino acid to increase the affinity of α-actinin for actin [18,42]. Similarly, the introduction of phosphorylation sites at this interface could be responsible for favoring the open state of CH1-CH2, thereby increasing the binding affinity for F-actin. Interestingly, rat α-actinin-2 K239 lies at the CH1-CH2 interface and is also found to be a ubiquitination site in this study. Other modifications like acetylation at K205, which became enriched with mechanical load, were not confirmed with western blots. This could be due to the marginally low levels of acetylation seen across the protein lysates. Nevertheless, the acetylation of residue K205 introduces a neutralization of a positively charged lysine that lies in close proximity to positively charged residues, which were previously identified to be involved in stabilization of PIP2 binding to α-actinin [20]. PIP2 increases α-actinin-2 opening of the ABD to favor binding of titin Zr-7 domains by the CaMD domains. This potential crosstalk between acetylation of ABD and altered PIP2 binding needs to be further studied.

Muscle energetics are tightly coupled to minimal levels of contractile protein [12], so that ubiquitination of α-actinin and of cellular lysates may assist in reduction of wasteful crossbridge cycling. Gain in K71 ubiquitination, seen with mechanical unloading, is located within a predicted degron [21]. Other modified sites, like S784p, K181ac, and T347p, could act as phosphodegron sites to initiate ubiquitination [51]. The ub-K48 motifs are not uniformly distributed throughout the cell but enriched at the outer annulus of cardiomyocytes where myofibrillogenesis or *de novo* formation of sarcomeres takes place [52], and this could be part of a mechanism to slow or diminish sarcomere assembly at the origin. In skeletal muscles, myosin mRNA concentrates at the myocyte periphery in the sub-sarcolemma annulus and in the myotendinous junction with prolonged muscle stretching [53]. The cell periphery of cardiac muscle cells is also the site where desmoplakin is located and mutations in this protein or destabilization of associated proteins leads to its ubiquitination and degradation [54]. In neurons, E3 ligases like TRIM9 and TRIM67 ubiquitinate the actin polymerase vasodilator-stimulated phosphoprotein (VASP) to reduce VASP filopodial tip localization and growth dynamics, affecting filopodial stability responsible for growth cone dynamics and axon guidance [55]. As such, a common cellular mechanism to slow cytoskeletal assembly may involve ubiquitination of actin-binding proteins at the cell periphery where cytoskeletal assembly takes place.

Within sarcomeres, the gain in ub-K48 signal is seen with chemical approach using Mava as well as with mechanical unloading, suggesting that both unloading paradigms target ongoing degradation. Interestingly, results here are the first to show a concentration of oligo-ub-K48 at the I-band, which might be related to other biological entities located in that region, such as ribosomes, proteasomes and mRNA [56–58]. Enriched ub-K48 signal at the I-band could be positioning ubiquitin-tagged proteins for local degradation by the proteasomes. Whole proteome characterization of the kinetics of degradation of proteins in mammalian cells identified two populations: one in which the kinetics of degradation were exponential, and a second population enriched by over-synthesized subunits of multiprotein complexes in which the kinetics of degradation had a fast rate of decay followed by a much slower rate [59]. It is plausible that when α-actinin and other structural proteins are dissociated, they undergo fast rates of degradation by being more susceptible to targeted degradation, but upon cross-linking with one another their protein degradation rates are slowed by being protected from ubiquitin-proteasome degradation. Thus, disassembly of sarcomeres triggered by unloading may permit access for poly-ubiquitin tagging of sarcomeric proteins and their subsequent degradation. These new findings may provide a mechanistic basis to explain earlier studies using isotope labeling and electron microscopy showing enhanced degradation when sarcomeres are disassembling in cardiac and skeletal muscle [8,60,61].

The new data here suggest that control of sarcomeric protein loss by unloading could be driven in part by dephosphorylation of α-actinin to weaken its binding to actin and permit unraveling of the thin filament bundling. This may allow localized ubiquitination and protein degradation to ensue. This may explain the earlier findings that unloaded sarcomeres have higher rates of protein degradation [12,62]. From the perspective of HCM, the goal is to discover regulatory nodes that control protein homeostasis via mechanosensation to reduce cell size when hypertrophy is the problem. Conversely, starting from a state of heart failure with reduced ejection fraction, hypocontractility, or long-term bed rest, the question is how cardiac muscle cell size can be preserved to increase contractile capacity and counteract atrophy. Certainly, novel therapies for these diseases might come from new understanding of biologic processes within cells. To this end, it is of utmost importance to understand the mechanisms by which cells regulate their structural protein inventories and how mechanical feedback alters this equilibrium.

## Materials and methods

The experimental workflow is described in Fig. 1. Additional details for Materials and methods are found in the Data S1.

### Neonatal rat ventricular myocytes

Neonatal rat ventricular myocytes were isolated and cultured as previously described [26,63]. After NRVRMs were maintained in 10% FBS overnight, serum-free media was used in all subsequent experiments. Near 60 pups were used across all the experiments in this work. All the animal procedures were approved by the Animal Care Committee at University of Illinois at Chicago.

### Chemical and mechanical loading and unloading

Two experimental paradigms of mechanical force stimulation were applied to cultured NRVMs to study the effects on sarcomere assembly or disassembly by anatomical, chemical, and biophysical changes. The first approach was an intrinsic stimulus elicited by drugs that modify myosin tension within the sarcomere. The second approach was an extrinsic stimulus from a 10% strain applied to the cardiomyocyte attachment substrate. NRVMs were treated with the myosin inhibitor Mava (1 μM, 6 h) or the myosin activator OM (0.5 μM, 6 h). The concentrations chosen were based on previous reports [64]. For external force generation, NRVMs were treated with 1 Hz cyclic strain for 24 h for loading (flex) as described previously [28], followed by 6 h rest to unload (flex-unflex), and compared to untreated cells (UT) that were not cyclically strained.

### Immunofluorescence

Fixed cell cultures in glass-bottom dishes or Flexcell membranes were blocked and stained with primary and secondary antibodies as described [65] for immunofluorescence (Zeiss Axio Observer.Z1) and confocal and super-resolution imaging (Zeiss LSM 880 with AiryScan). Cell cultures treated with OM and Mava were fixed at 0, 2, 4, 6, and 12 h. Cultures unflexed (UF), undergoing 24 h of cyclic straining (F), or 24 h strain followed by 6 h of recovery (F-UF) were fixed at the term of the protocol. Refer to Table 1 for antibodies used.

### Fluorescence recovery after photobleaching

Cardiac muscle cells infected with adenoviral α-actinin-YFP and CapZ-GFP were photobleached (> 50% fluorescence decay) at single z-discs (ROI ~ 2 × 2 μm) and the fluorescence intensity was monitored over a period of 12 min (5 s time intervals) to derive the kinetic rate of fluorescence recovery, *k*_*FRAP*_ as previously described [40,66].

### Mass spectrometry

Trypsinized SDS/PAGE gel bands (250 ng) were loaded into a C18 Aurora column (IonOpticks) for separation and analysis by mass spectrometry (Bruker timsTOF Pro). Data were acquired using a data-dependent acquisition PASEF method previously described [67]. Mass spectrometry data was analyzed with PEAKS Studio 10.6 (build 20 201 015, Bioinformatics Solutions Inc., Waterloo, ON, Canada) against a Uniprot *Rattus norvegicus* database with 36 180 entries. PTMs found in each peptide with an Ascore of > 13 (Table S2, S3) were selected for analysis as described previously [68].

### Western blots

Lysed cardiomyocytes were subjected to SDS/PAGE and transferred to PVDF membranes (Millipore Immobilion^®^-P^SQ^) as described [69,70]. After blocking with dry milk, primary and secondary antibodies were incubated, and membranes were imaged (Bio-Rad Chemidoc MP, Hercules, CA, USA). Refer to Table 1 for antibodies used.

### Statistical analysis

Statistical analyses were conducted using RStudio (version 2022.02.0443, Posit, PBS, Boston, MA, USA). Bar graphs and histograms are represented as mean standard deviation (SD) indicating the number of replicates made. Multiple comparisons are tested by one-way ANOVA. *Post hoc* testing uses Tukey’s honest significance test. The null hypothesis comparing two mean values was rejected at a significance level of 1 – α > 0.95. Data normal distribution and skewness was evaluated by quantile-quantile plots. In this work, *N* indicates the number of independent primary cardiomyocyte cultures made, which each consists of 20 neonatal hearts or more.

## Supplementary Material

Supplementary_Material **Data S1**. Materials and methods.
**Fig. S1**. Mavacamten causes sarcomere disassembly in human stem cell-derived cardiomyocytes.
**Fig. S2**. Validation of the poly-UbK48 antibody sensitivity to accumulation of ubiquitinated proteins.
**Fig. S3**. Relative abundance of post-translational modifications in α-actinin-2.
**Fig. S4**. Whole Ponceau S, α-actinin, and Acetylation Western blot panel.
**Fig. S5**. Whole Ponceau S, α-actinin, and Phospho-Serine Western blot panel.
**Fig. S6**. Whole Ponceau S, α-actinin, and Phospho-Threonine Western blot panel.
**Fig. S7**. Whole Ponceau S, α-actinin, and oligo-UbK48 Western blot panel.
**Fig. S8**. Whole Ponceau S, GAPDH, and phospho-PKA substrate Western blot panel.
**Fig. S9**. ERK1/2 signaling Western blot panel.
**Fig. S10**. PKC signaling Western blot panel.
**Fig. S11**. FAK signaling Western blot panel.
**Table S1**. Predicted phosphorylation sites and kinases for α-actinin-2 based on the rat ACTN2.
**Table S2**. Mass spectrometry dataset for chemically unloaded samples.
**Table S3**. Mass spectrometry dataset for mechanically unloaded samples.

## Figures and Tables

**Table 1.** List of antibodies for western blot analysis.

Primary antibody	Dilution factor

MS anti-sarcomeric α-actinin EA-53 (Abcam #9465, Waltham, MA, USA)	1 : 1000
RB anti-acetyl-Lys (Cell Signaling Tech #9441S, Danvers, MA, USA)	1 : 1000
RB anti-phospho-Ser (Abcam #9332)	1 : 1000
RB anti-poly-K48-ubiquitin (Abcam #140601)	1 : 500
RB Phospho-PKA Substrate (RRXS*/T*) (100G7E) (Cell Signaling Tech #9624)	1 : 1000
RB anti-phospho-FAK (Tyr397) (Invitrogen #44–624G, Waltham, MA, USA)	1 : 500
MS anti-FAK (Invitrogen #34Q36)	1 : 1000
RB anti-phospho-PKCepsilon (S729) (Abcam #63387)	1 : 500
RB anti-PKC (Abcam #179522)	1 : 1000
Phospho-p44/42 MAPK (Erk1/2) (Thr202/Tyr204) (Cell Signaling Tech #9101)	1 : 1000
RB p44/42 MAPK (Erk1/2) (Cell Signaling Tech #9102)	1 : 1000
Anti-Histone H2B antibody (Cell Signaling Tech #12364)	1 : 2000
RB Acetylated-Lysine MultiMab (Cell Signaling Tech #9814)	1 : 1000
Anti-RB (Goat IgG (H + L)) Secondary Antibody, DyLight™ 800 4X PEG (Invitrogen #SA5–35571)	1 : 5000
Anti-MS StarBright Blue 700 (Goat IgG) (Bio-Rad #12004158, Hercules, CA, USA)	1 : 5000

## Data Availability

Additional data supporting the results of this study can be found in the Supporting Information or are available from the corresponding authors upon reasonable request.

## Abbreviations

ABD: actin binding domain
CaMD: calmodulin-like domain
ERK1/2: extracellular signal-regulated kinase 1/2
F: flexed
FAK: focal adhesion kinase
FRAP: fluorescence recovery after photobleaching
F-UF: flexed-unflexed
GFP: green fluorescent protein
HCM: hypertrophic cardiomyopathy
LVAD: left ventricular assist device
Mava: mavacamten
NRVM: the neonatal rat ventricular myocyte
OM: omecamtiv mecarbil
PIP2: phosphatidylinositol 4,5-bisphosphate
PKA: protein kinase A
PKC: protein kinase C
PTM: post-translational modification
UF: unflexed
UT: untreated
YFP: yellow fluorescent protein
