# Supplementary material for: Cardiomyocyte external mechanical unloading activates modifications of α-actinin differently from sarcomere-originated unloading
Source: FEBS J. Author manuscript; Available in PMC 2024 Jul 29. (PMC11285078; doi:10.1111/febs.16925)
Supplement: Supplementary_Material — Data S1. Materials and methods. Fig. S1. Mavacamten causes sarcomere disassembly in human stem cell-derived cardiomyocytes. Fig. S2. Validation of the poly-UbK48 antibody sensitivity to accumulation of ubiquitinated proteins. Fig. S3. Relative abundance of post-translational modifications in α-actinin-2. Fig. S4. Whole Ponceau S, α-actinin, and Acetylation Western blot panel. Fig. S5. Whole Ponceau S, α-actinin, and Phospho-Serine Western blot panel. Fig. S6. Whole Ponceau S, α-actinin, and Phospho-Threonine Western blot panel. Fig. S7. Whole Ponceau S, α-actinin, and oligo-UbK48 Western blot panel. Fig. S8. Whole Ponceau S, GAPDH, and phospho-PKA substrate Western blot panel. Fig. S9. ERK1/2 signaling Western blot panel. Fig. S10. PKC signaling Western blot panel. Fig. S11. FAK signaling Western blot panel. Table S1. Predicted phosphorylation sites and kinases for α-actinin-2 based on the rat ACTN2. Table S2. Mass spectrometry dataset for chemically unloaded samples. Table S3. Mass spectrometry dataset for mechanically unloaded samples. [file NIHMS2008560-supplement-Supplementary_Material.pdf]

Supplementary Information for

Cardiomyocyte external mechanical unloading activates modifications of α-actinin differently from sarcomere-originated unloading

Christopher Solís*, Chad M. Warren, Kyle Dittloff, Elisabeth DiNello, R. John Solaro, and Brenda Russell*
Department of Physiology and Biophysics, Center for Cardiovascular Research, University of Illinois at Chicago, Chicago, IL, USA
***Email:** csolisoc@uic.edu, russell@uic.edu

Supplementary Materials and Methods

Aminosilanized Flexible Membranes. To modify 6-well BioFlex membranes with fibronectin, membranes were treated with 5% (v/v) of (3-Aminopropyl) triethoxysilane (Millipore Sigma, #440140) in 95% (v/v) ethanol for 10 min at room temperature. Membranes were treated with 100% ethanol, aspirated, and dried in the oven at 57ºC for 20 min. Membranes were washed with 95% (v/v) ethanol, then PBS buffer, Milli-Q water and treated with 0.01 mg/mL of fibronectin (Corning, # 356008) for 2 hr at 37ºC. After incubation, membranes were washed with autoclaved Milli-Q water and sterilized with a UV lamp for 20 min.

Neonatal Rat Ventricular Myocyte Cultures. All animal procedures were conducted under protocols approved by the university Animal Care Committee. Neonatal rat ventricular myocytes (NRVMs) were obtained as previously described (Solís & Russell, 2019). Briefly, hearts obtained from 1–2 day old Sprague-Dawley rats were minced and digested with collagenase type II (Worthington Biochemical Corporation, #CLS-2). After incubation at 37°C for 10 min with agitation, NRVMs in suspension (10 ml) were collected and added to 4ºC fetal bovine serum (10 ml). This process was repeated until the tissue was completely digested (usually between five and six repetitions). NRVMs were filtered through a 70 µm nylon sieve, centrifuged, and resuspended in plating media. Myocytes were plated on glass-bottom dishes modified with fibronectin or BioFlex® Culture Plates (Flexcell® International Corporation, # BF-3001U) modified with fibronectin at a density of $1.6 \times 10^5/cm^2$ in plating media (68% DMEM, 17% Medium 199, 10% horse serum, and 5% fetal bovine serum) for one day before replacing media with maintenance media (80% DMEM and 20% Medium 199).

Human-induced pluripotent stem cell-derived cardiomyocyte (iPSC-CM) culture. Human iPSC-CM from a male donor was purchased from Fujifilm Cellular Dynamics Intl. (iCell Cardiomyocytes, cat# R1105). Upon thaw, cells were plated on fibronectin or TTR-coated substrates and maintained for 7 days before assay as described previously (Dittloff et al., 2022).

Adenoviral Infection. Recombinant adenoviruses for α-actinin-2-YFP and CapZβ1-GFP were kindly provided by Drs. Joseph, Jean Sanger (Wang et al., 2005) and Dr. Jody Martin (Hartman et al., 2009). NRVMs were infected 1 day after isolation with α-actinin-2-YFP (MOI 20) or CapZβ1-GFP (MOI 20) for 60 min at 37°C in maintenance media. Viral media was replaced with virus-free media, and cells were left undisturbed for 18-24 h prior to use in the experiment.

Drug Treatment. After replacing serum-containing media with serum-free media, omecamtiv mecarbil (Selleck Chemicals, #S2623) and mavacamten (MedChemExpress, #HY-109037) stored in DMSO were added at 0.5 µM and 1 µM respectively in maintenance media.

Mechanical Strain. Two days after plating NRVMs on 6-well BioFlex membranes, cultures were strained at 10% elongation biaxially and 1 Hz sinusoidally for 1 hr with a Flexcell Strain Unit (model FX-4000, Flexcell International, Burlington, NC), as described previously (Senyo et al.,

2007). The load modalities are summarized in Fig. 1 showing three conditions applied to NRVM cultures: unflexed (UF), 24 hr flexed (F), and 24 hr flexed followed by a 6 hr unflexed period (F-UF). The F condition was started 6 hr after the initiation of the F-UF condition to match the end time of all conditions. Cell lysis for western blot and mass spectrometry analysis or fixing for microscopy proceeded after the flexing protocol.

Subcellular Fractionation and Immunofluorescence. For whole-cell fixing, NRVM cultures were fixed with -20ºC methanol for 15 min at -20ºC and washed three times with PBS for 5 min each. For subcellular fractionation, NRVM cultures were treated as described previously (Boateng et al., 2007). Briefly, cytosol, sequential cytosol and membrane, or sequential cytosol, membrane, and nuclear extraction buffers (ProteoExtract Subcellular Proteome Extraction kit, EMD Millipore, # 539790) were added to extract the respective cellular compartments. In addition, protease (Calbiochem, #539131), phosphatase (EMD Millipore, #524625), deacetylase (Santa Cruz Biotechnology, # 362323), and deubiquitinase (Selleck Chem, #S7130) inhibitors were added to each buffer. After extraction of each cellular compartment, cell cultures were fixed as described for whole-cell culture fixing. After fixing and PBS washing, primary antibodies diluted in 5% (w/v) BSA and 0.1% (v/v) tween-20 were applied to cell cultures and incubated overnight at 4ºC in a rocking platform shaker. Next, dishes were washed three times with PBS and the secondary antibodies diluted in 5% (w/v) BSA and 0.1% (v/v) tween-20 were applied to cell cultures and incubated for 1 hr at room temperature. After completion, NRVM cultures were washed four times with PBS and the bound antibodies were fixed with a formaldehyde solution (10%) for 10 min at room temperature before washing three times with PBS and applying mounting media (Vector Laboratories, # H-1200) for imaging. For a list of antibodies applied and respective dilution factors refer to Table S1.

Image Analysis. Quantitative image analysis was conducted using Cell Profiler and MATLAB. The codes are available in a public repository (https://github.com/chsolis).

Immunoprecipitation and Western Blots. For immunoprecipitation assays, NRVM cultures were lysed with RIPA buffer containing protease, phosphatase, deacetylase, and deubiquitinase inhibitors, incubated for 10 min at 4ºC and stored at -20ºC. Individual samples were immunoprecipitated using 10 µg of Dynabeads™ with Protein G (Fisher Scientific/Invitrogen 10007D) crosslinked with 50 mM dimethyl pimelimidate HCl (in 0.2 M triethanolamine pH 8.2) to anti-α-actinin (2 µl) (50 µl) antibody following to the manufacturer's protocol. After washing, samples were eluted in 40µl of 2x Laemmli buffer with β-mercaptoethanol. Samples from the same treatment were pooled and concentrated by precipitating with four volumes of -20ºC acetone. Samples were incubated overnight, centrifuged at 14,000 rcf, and the precipitates were resuspended in 8M urea, 2M thiourea, 0.05M Tris pH 6.8, 75mM DTT, 3% (w/v) SDS, 0.005% (w/v) bromophenol blue (ISB) buffer (Fritz et al., 1989) for SDS-PAGE separation.

Samples were then prepared for Western blot by adding 4X Laemmli sample buffer (BioRad, Inc., Hercules, CA). Protein extracts from sarcomeric subcellular fractions were resolved by SDS/PAGE and transferred to polyvinyl difluoride membrane similar to previously described (Batra et al., 2021) with some minor modifications. Electrophoretic transfer was conducted in 10 mM CAPS pH 11.0 buffer for 2 hr at 20 constant volts. Immunoblots were blocked for 1 hr in 5% nonfat milk in tris-buffered saline, pH 7.5, containing 0.1% Tween-20 (TBST) at room temperature and washed three times for 10 min each in TBST. Immunoblots were then probed in primary antibody overnight at 4ºC. Antibodies were diluted in 2.5% bovine serum albumin in TBST. Refer to table S1 for a complete list of antibodies.

For multiplex Western blots, gels were transferred to WesternBright PVDF-FL membranes (Advansta #L-08007-001) using the same electrophoretic transfer settings as conventional polyvinyl difluoride membranes. Membranes were blocked with AdvanBlock-Fluor (Advansta #R-03729-E10) for 1 hr at room temperature. Primary and secondary antibodies were diluted in AdvanBlock-Fluor and incubated as conventional polyvinyl difluoride membranes with the exception that fluorescently labeled secondary antibodies were protected from light exposure.

After washing the membrane with TBST, a final wash with ddH2O was made and membranes were dried in the dark before imaging with a Bio-Rad Chemidoc MP with auto-capture settings for two-color imaging.

Confocal and Near Super-Resolution Microscopy. Fixed samples were imaged with a Zeiss LSM 880 confocal microscope with AiryScan imaging (Carl Zeiss, Jena, Germany). A Plan-Apochromat 63x/1.4 Oil DIC M27 objective was used to image at an x-y-z resolution of 140 nm by 140 μm by 400 nm.

Fluorescence Recovery After Photobleaching. FRAP measurements were made with a confocal light scanning microscope (LSM 880, Carl Zeiss) with controlled temperature (37°C) and CO2 (5%). A single sarcomeric region of interest (ROI) was selected for photobleaching, while a contiguous sarcomere was used as the reference. After an ROI was photobleached, the light intensity in the ROI and the reference were monitored for up to 900 s at 5 frames per second. The characteristic intensity recovery rate, kFRAP, was determined by the equation $I(t) = 1 - C_1 e^{-koff1t} - C_2 e^{-koff2t}$, where I is the intensity in a reference-weighed ROI, C1 and C2 are preexponential constants, and koff1 and koff2 are the kinetic constants. FRAP kinetics can be modeled with two phases, but only the simple constant was used here to provide the most general information. The amplitude-weighted kinetic constant kFRAP was determined using the following formula kFRAP = $C_1 k_{off1} + C_2 k_{off2}$.

Sample preparation for mass spectrometry
α-actinin was immunoprecipitated from NRVMs as described above to enrich for the protein to better identify post-translational modifications (PTMs). The bound protein was eluted with 2X Laemmli buffer supplemented with β-mercaptoethanol as recommended by the manufacturer (Bio-Rad #1610737) from 8 pooled separate experiments to allow for enough enriched protein. The proteins were eluted from Dynabeads (Invitrogen) and were then separated in 12% SDS-PAGE precast gels (Bio-Rad #4561043) and stained with Coomassie G-250 Bio-Safe (Bio-Rad #1610786). The bands of interest were cut out of the gel and an in-gel trypsin gold digest was performed following the manufacturer's recommendations (Promega #V5280) with minor modifications.

Briefly, the gel slices were destained twice with 100 mM NH4HCO3 pH 8.2, 50% (v/v) acetonitrile for 45 min each at 37°C in a Thermomixer C (Eppendorf) set at 600 RPM. Next, the gel pieces were dehydrated with 100% (v/v) acetonitrile, and the slices were pre-incubated with 25uL of 20ug/ml of trypsin gold (Promega #V5280) in digestion buffer (40 mM NH4HCO3 pH 8.2, 10% (v/v) acetonitrile) at room temperature for 1 hr to rehydrate. Digestion buffer was added to cover the gel pieces and incubated for 15 hr and 37°C in a Thermomixer C (Eppendorf) set at 600 RPM.

After the peptides were digested, they were eluted from the gel slices with 150 μL of water for 10 min at room temperature and combined with two subsequent elutions of 50% (v/v) acetonitrile, 5% (v/v) formic acid for 60 min at room temperature with continuous vortex shaking. The eluted peptides were dehydrated with speed vacuum and resuspended in 25μL of 20% (v/v) acetonitrile, 0.1% (v/v) formic acid to determine the amount of peptide via a colorimetric peptide assay (Thermo Scientific #23275). Finally, the peptides were speed vac down to dryness and stored at -80°C.

Liquid chromatography tandem mass spectrometry
Peptides were resuspended in 0.1% (v/v) formic acid and 250ng directly loaded onto a 75um x 25 cm 1.6um C18 Aurora column (IonOpticks) with Captive Spray emitter. Peptides were separated using a nanoElute UHPLC (Bruker) at 400 nl/min. Solvent A composition was 0.1% (v/v) formic acid in water, Solvent B was 0.1% (v/v) formic acid in acetonitrile. Linear step gradient conditions were 0 to 60 min (2%B to 17%B), 60 min to 90 min (17% to 25%B), 90 to 100 min (25% to 38%B), 100 to 110 min (38% to 90%B), 110 to 120 (90%). Eluting peptides were then further separated using TIMS (trapped ion mobility spectrometry) on a Bruker timsTOF Pro mass

spectrometer. Mass spectrometry data were acquired using a data-dependent acquisition PASEF method previously described (Meier et al., 2018). The acquisition scheme used was 100 ms accumulation, 100 ms PASEF ramp (at 100% duty cycle) with up to 10 PASEF MS/MS scans per topN acquisition cycle. The capillary voltage was set at 1700V, capillary gas temp 200C. The target value was set at 20,000 a.u. with the intensity threshold set at 5000 a.u. The m/z range surveyed was between 100 to 1700. Precursor ions for PASEF-MS/MS were selected in real-time from a TIMS-MS survey scan using a non-linear PASEF scheduling algorithm. The polygon filter (200 to 1700 m/z) was designed to cover ions within a specific m/z and ion mobility plane to select multiply charged peptide features rather than singly charged background ions. The quadrupole isolation width was set to 2 Th for m/z < 700 and 3 Th for m/z > 700.

Mass spectrometric data analysis

The data-dependent acquired timsTOF Pro data were analyzed with PEAKS Studio 10.6 build 20201015 with PEAKS IM, PEAKS Platform, and PEAKS Q modules. The data were searched against a Uniprot Rat norvegicus database downloaded on Jan 21, 2021, with 36,180 entries along with a common repository of adventitious proteins (cRAP) downloaded on March 3, 2019, from the global proteome machine which contained 116 contaminant proteins that were excluded post searching. Searches were configured for specific trypsin with a max of 2 missed cleavages, 20 PPM monoisotopic precursor mass tolerance, 0.1Da fragment ion mass tolerance. The PEAKS search contained no fixed modifications, but the variable modifications were phosphorylation (STY), acetylation (K, N-term), ubiquitination (TSCK) along with another 306 built-in default post-translational modifications within the PEAKS PTM module, however, these modifications were excluded post searching. The protein threshold score was set at -10logP ≥ 20 and the peptide threshold score of -10logP ≥ 15.6, and the false discovery rate (5%) for the peptides was based on a fusion decoy where the target and decoy sequences of the same protein are concatenated. A minimum of at least two unique peptides were required for the identification of a protein. To quantify the confidence of a PTM site assignment an Ascore of > 13 (P=0.05) was considered 95% certain (Jedrychowski et al., 2011) and thus was used as filter criteria for any PTM's identified post searching. A maximum number of PTMs were set to 5 per peptide. The PTM quantitation was done within PEAKS Studio with PTM Profiler as previously described (Han et al. 2006) to report relative areas of modified vs unmodified (Fig. S4). Only the α-actinin-2 protein was used post search and all samples had at least 89% sequence coverage of α-actinin-2. The mass spectrometry data have been deposited in the ProteomeXchange Consortium (http://proteomecentral.proteomexchange.org) via the MassIVE partner repository (https://massive.ucsd.edu/ProteoSAFe/static/massive.jsp) with the dataset identifier MSV000091010. The URL to the dataset is ftp://massive.ucsd.edu/MSV000091010/. Post search analysis with R Studio (v. 2022.02.0 Build 443) was done to quantify the fold-changes of post-translational changes across single residues and the spreadsheets can be found in the supplementary tables S2 and S3.

Statistical analysis

Statistical analyses were conducted using RStudio (version 2022.02.0 443). Bar graphs and histograms are represented as mean ± standard deviation (sd) indicating the number of replicates made. Multiple comparisons are tested by one-way ANOVA. Post hoc testing uses Tukey's honest significance test. The null hypothesis comparing two mean values was rejected at a significance level of 1−α > 0.95. Data normal distribution and skewness was evaluated by quantile-quantile plots.

References

Batra, A., Warren, C. M., Ke, Y., McCann, M., Halas, M., Capote, A. E., Liew, C. W., Solaro, R. J., & Rosas, P. C. (2021). Deletion of P21-activated kinase-1 induces age-dependent increased visceral adiposity and cardiac dysfunction in female mice. *Molecular and Cellular Biochemistry*, *476*(3), 1337–1349. https://doi.org/10.1007/s11010-020-03993-3

Boateng, S. Y., Belin, R. J., Geenen, D. L., Margulies, K. B., Martin, J. L., Hoshijima, M., de Tombe, P. P., & Russell, B. (2007). Cardiac dysfunction and heart failure are associated with abnormalities in the subcellular distribution and amounts of oligomeric muscle LIM protein. *Am J Physiol Heart Circ Physiol*, *292*(1), H259-69. https://doi.org/10.1152/ajpheart.00766.2006

Dittloff, K. T., Spanghero, E., Solís, C., Banach, K., & Russell, B. (2022). Transthyretin deposition alters cardiomyocyte sarcomeric architecture, calcium transients, and contractile force. *Physiological Reports*, *10*(5), e15207. https://doi.org/10.14814/phy2.15207

Fritz, J. D., Swartz, D. R., & Greaser, M. L. (1989). Factors affecting polyacrylamide gel electrophoresis and electroblotting of high-molecular-weight myofibrillar proteins myofibrillar proteins. *Analytical Biochemistry*, *180*(2), 205–210. https://doi.org/10.1016/0003-2697(89)90116-4

Hartman, T. J., Martin, J. L., Solaro, R. J., Samarel, A. M., & Russell, B. (2009). CapZ dynamics are altered by endothelin-1 and phenylephrine via PIP2- and PKC-dependent mechanisms. *Am J Physiol Cell Physiol*, *296*(5), C1034-9. https://doi.org/10.1152/ajpcell.00544.2008

Jedrychowski, M. P., Huttlin, E. L., Haas, W., Sowa, M. E., Rad, R., & Gygi, S. P. (2011). Evaluation of HCD- and CID-type Fragmentation Within Their Respective Detection Platforms For Murine Phosphoproteomics *. *Molecular & Cellular Proteomics*, *10*(12). https://doi.org/10.1074/mcp.M111.009910

Meier, F., Brunner, A.-D., Koch, S., Koch, H., Lubeck, M., Krause, M., Goedecke, N., Decker, J., Kosinski, T., Park, M. A., Bache, N., Hoerning, O., Cox, J., Räther, O., & Mann, M. (2018). Online Parallel Accumulation–Serial Fragmentation (PASEF) with a Novel Trapped Ion Mobility Mass Spectrometer*. *Molecular & Cellular Proteomics*, *17*(12), 2534–2545. https://doi.org/10.1074/mcp.TIR118.000900

Senyo, S. E., Koshman, Y. E., & Russell, B. (2007). Stimulus interval, rate and direction differentially regulate phosphorylation for mechanotransduction in neonatal cardiac myocytes. *FEBS Lett*, *581*(22), 4241–4247. https://doi.org/10.1016/j.febslet.2007.07.070

Solís, C., & Russell, B. (2019). CapZ integrates several signaling pathways in response to mechanical stiffness. *The Journal of General Physiology*, *151*(5), 660–669. https://doi.org/10.1085/jgp.201812199

Wang, J., Shaner, N., Mittal, B., Zhou, Q., Chen, J., Sanger, J. M., & Sanger, J. W. (2005). Dynamics of Z-band based proteins in developing skeletal muscle cells. *Cell Motil Cytoskeleton*, *61*(1), 34–48. https://doi.org/10.1002/cm.20063

Supplementary Figures and Tables

Table S1. Predicted phosphorylation sites and kinases for α-actinin-2 based on the rat ACTN2. NCBI Reference Sequence: NP_001163796.1.

Motif	Position	Residue	Peptide	Predicted Kinase (NetPhos 3.1 score*)
ABD	43	Thr	QQRKTFTAW	PKC (0.732), PKA (0.526),
	50	Ser	--	cdc2 (0.461)
	116	Ser	VKLVSIGAE	Unspecified (0.846)
	147	Ser	IQDISVEET	Unspecified (0.995), CKII (0.540)
	237	Thr	DIVNTPKPD	Unspecified (0.990), p38MAPK (0.514)
Rod	308	Thr	LENRTPEKT	p38MAPK (0.537)
	347	Thr	--	--
	363	Ser	--	--
	369	Ser	--	--
	429	Tyr	--	--
	433	Ser	YESASLTEV	Unspecified (0.976), CKII (0.514), cdc2 (0.505)
	574	Ser	--	--
	599	Tyr	SSNPYSTVT	Unspecified (0.892), INSR (0.561)
CaMD	784	Ser	--	--
	822	Thr	MTRETADTD	Unspecified (0.622)

*https://services.healthtech.dtu.dk/services/NetPhos-3.1/

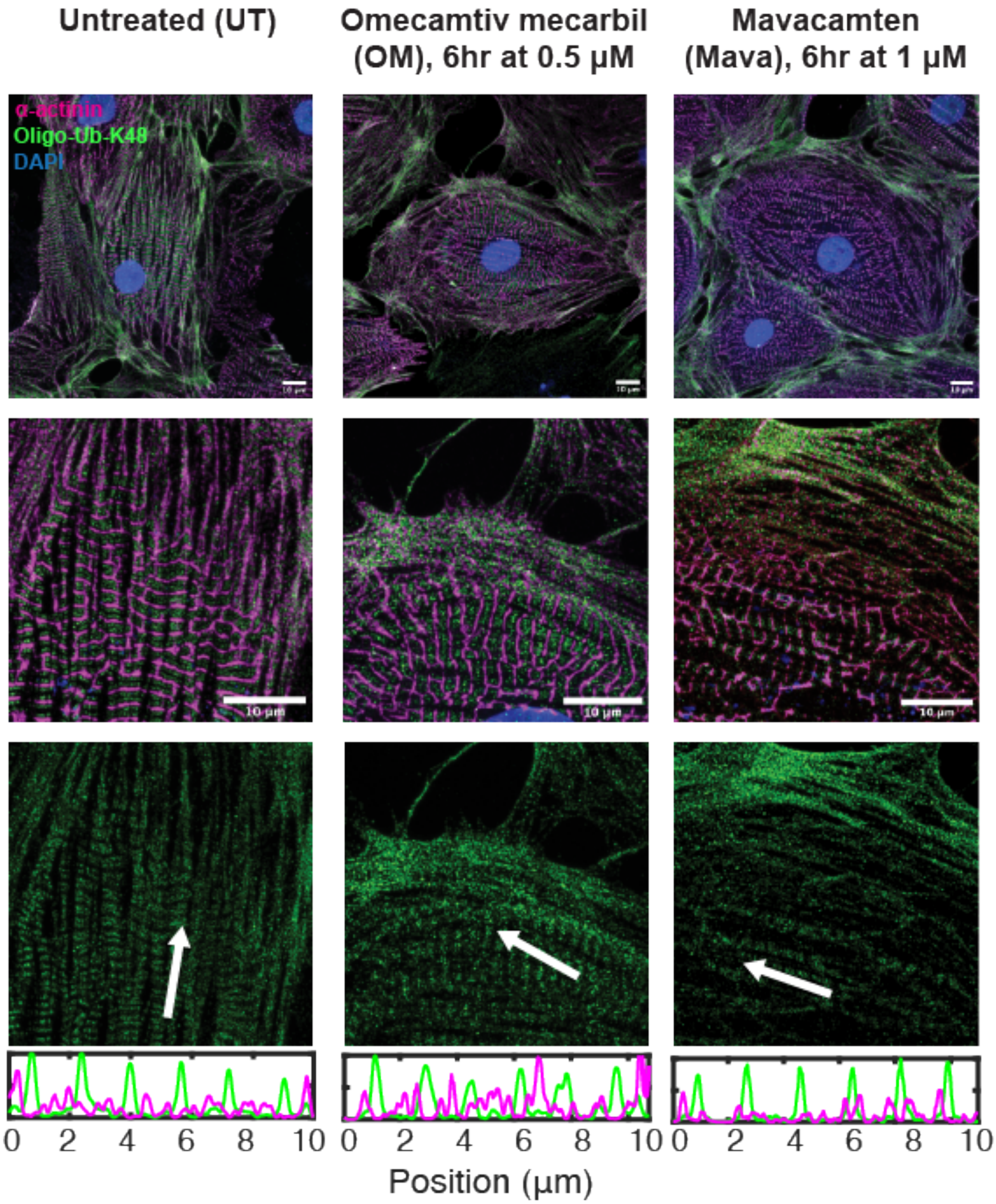

Figure. S1. Mavacamten causes sarcomere disassembly in human stem cell-derived cardiomyocytes. Immunofluorescence stains of untreated (UT) hiPSC-CMs are compared with hiPSC-CMs treated with omemcamtiv mecarbil (OM, 0.5 μM) or mavacamten (Mava, 1 μM) after fixing and staining with α-actinin (magenta) and oligo-Ub-K48. White arrows 10 μm long show direction and fluorescence intensity profile of ubiquitin-K48 (green) and α-actinin (magenta). Images were captured via super-resolution confocal AiryScan microscopy (scale, 10μm).

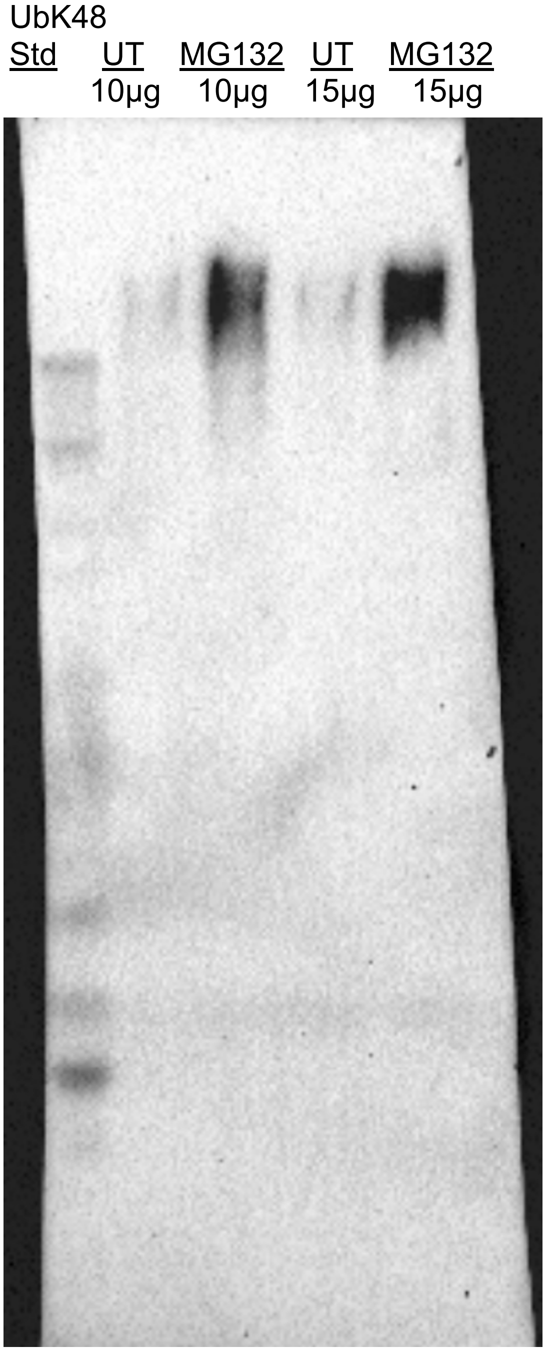

Figure S2. Validation of the poly-UbK48 antibody sensitivity to accumulation of ubiquitinated proteins. NRVMs were untreated (UT) or treated with 10 µM of MG-132 for 6 hr and the cell lysates were analyzed by western blots using the anti-oligo-Ub-K48 as a readout. Untreated (UT) and treated (MG132) samples were loaded at 10 and 15 µg.

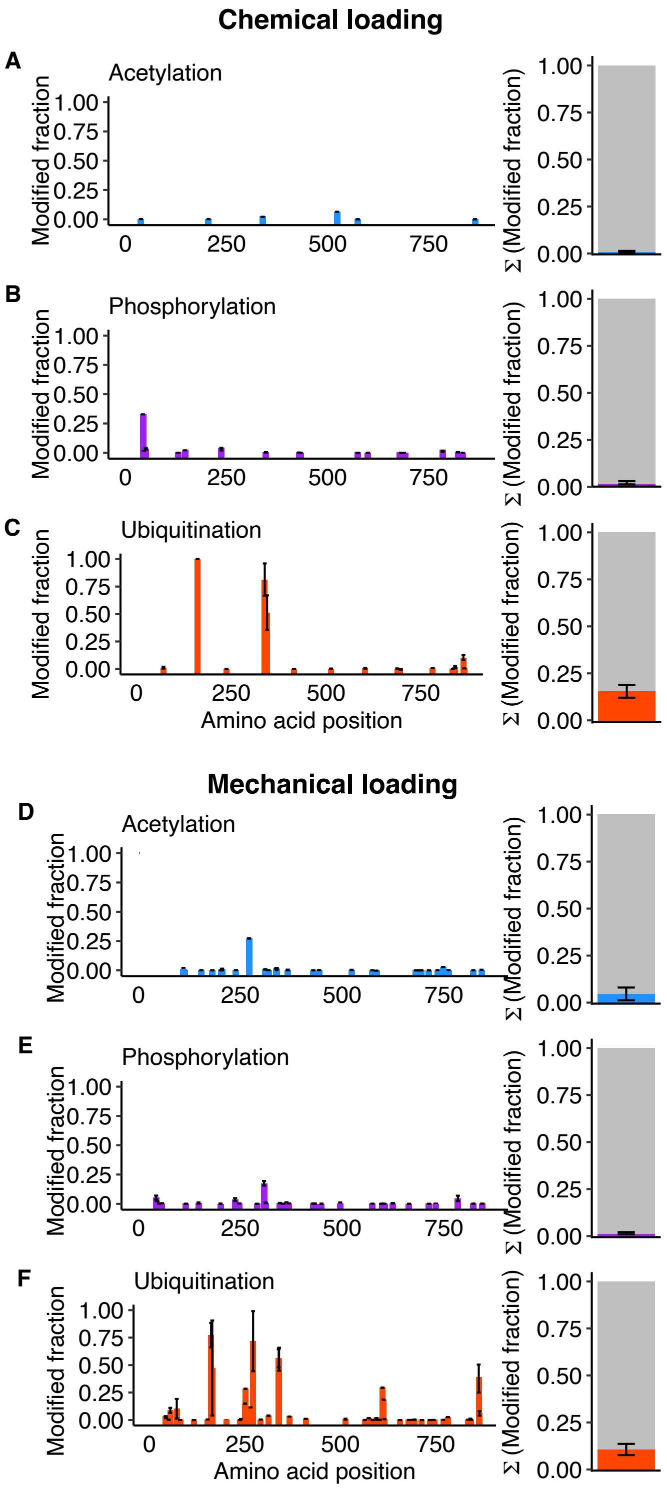

Figure. S3. Relative abundance of post-translational modifications in α-actinin-2. Relative abundance of modified residues, defined as the fraction of modified residues vs. unmodified (i.e. modified fraction = modified/(modified + unmodified) was calculated for the chemical (A-C) and the mechanical loading (D-F). The three major modifications quantified include acetylation (A, D), phosphorylation (B, E), and ubiquitination (C, F). Data are presented as mean ± S.D. (N = 3 cell cultures).

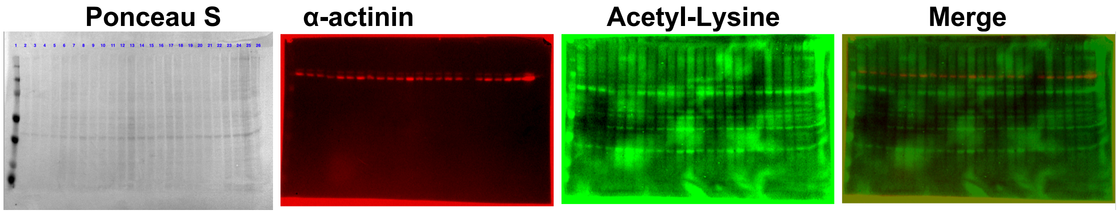

Figure S4. Whole Ponceau S, α-actinin, and Acetylation Western blot panel.

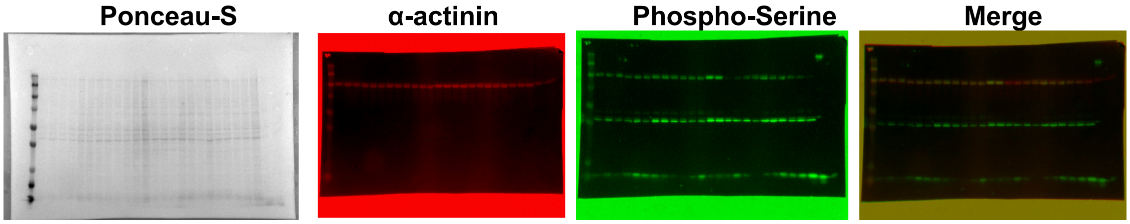

Figure S5. Whole Ponceau S, α-actinin, and Phospho-Serine Western blot panel.

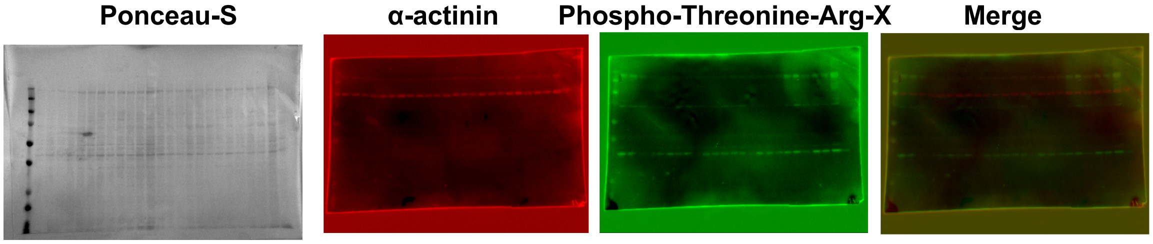

Figure S6. Whole Ponceau S, α-actinin, and Phospho-Threonine Western blot panel.

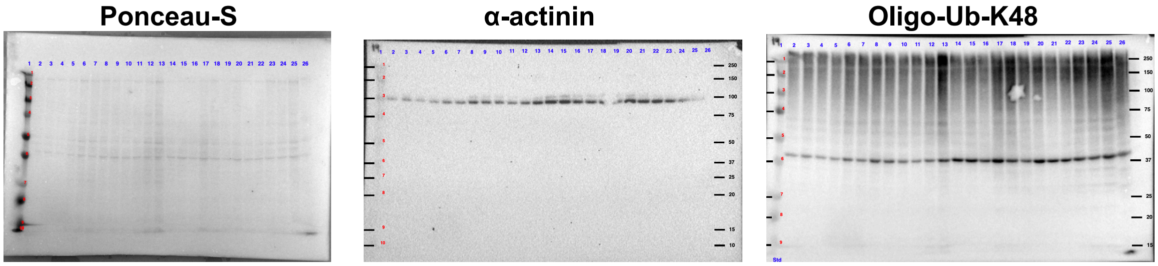

Figure S7. Whole Ponceau S, α-actinin, and oligo-Ub-K48 Western blot panel.

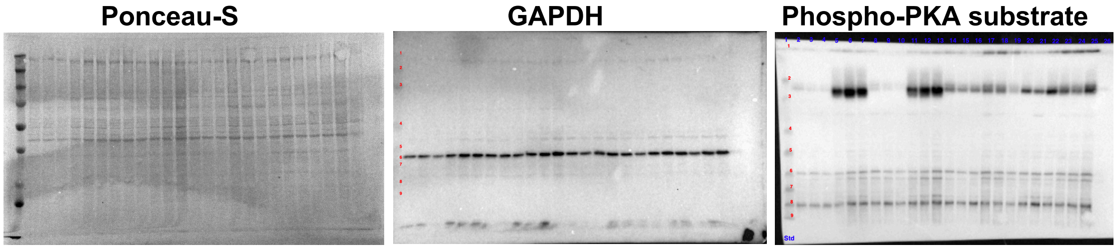

Figure S8. Whole Ponceau S, GAPDH, and phospho-PKA substrate Western blot panel.

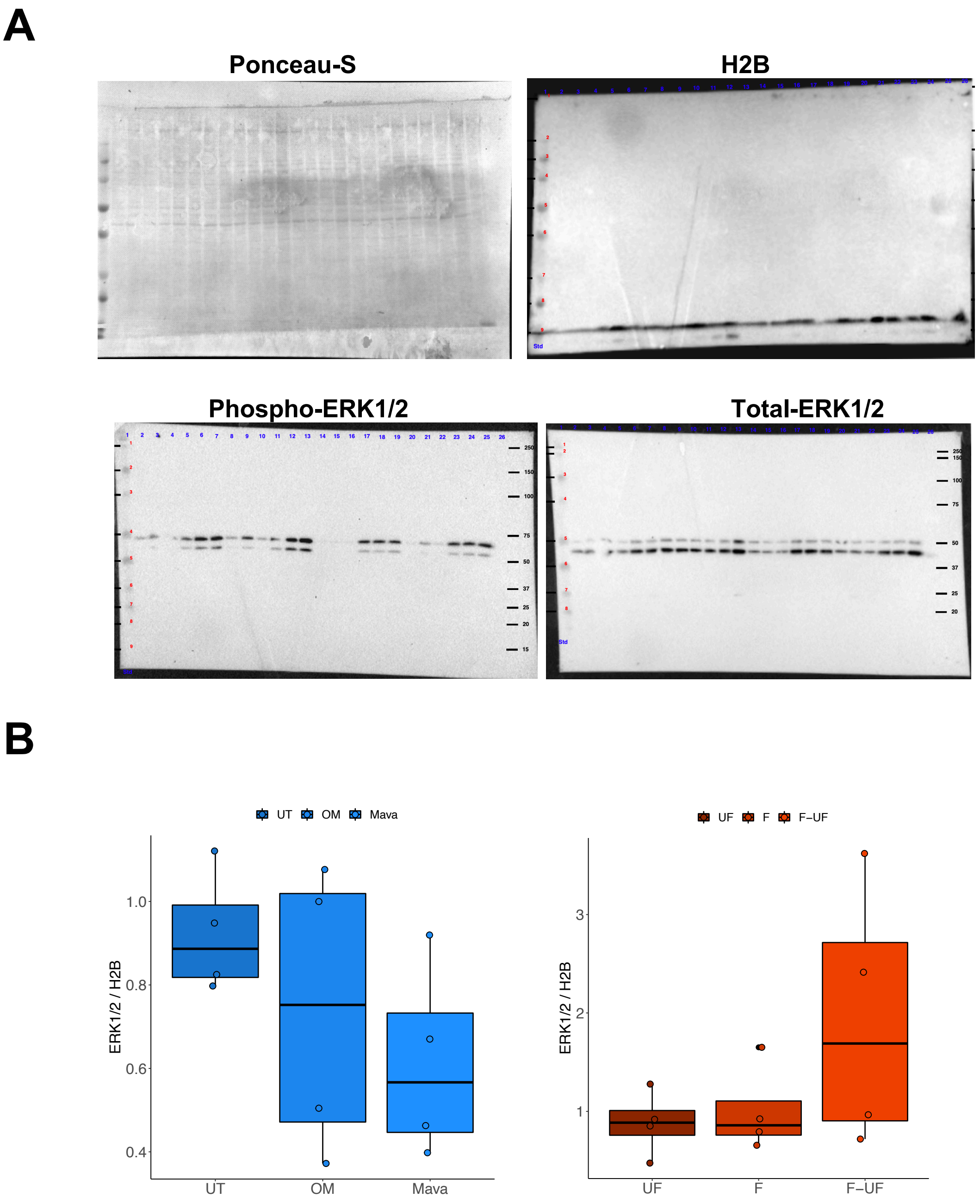

Figure S9. ERK1/2 signaling Western blot panel. (A) Whole Ponceau S, H2B, phospho-ERK, and total-ERK1/2 Western blots. (B) Quantification of total ERK1/2 to H2B for the chemical unloading model composed by untreated (UT), omecamtiv mecarbil (OM), and mavacamten (Mava) (*left*); and the mechanical unloading model composed by unflexed (UF), flexed (F), and flexed-unflexed (F-UF) samples (*right*). Data is presented as mean ± SD (N = 4).

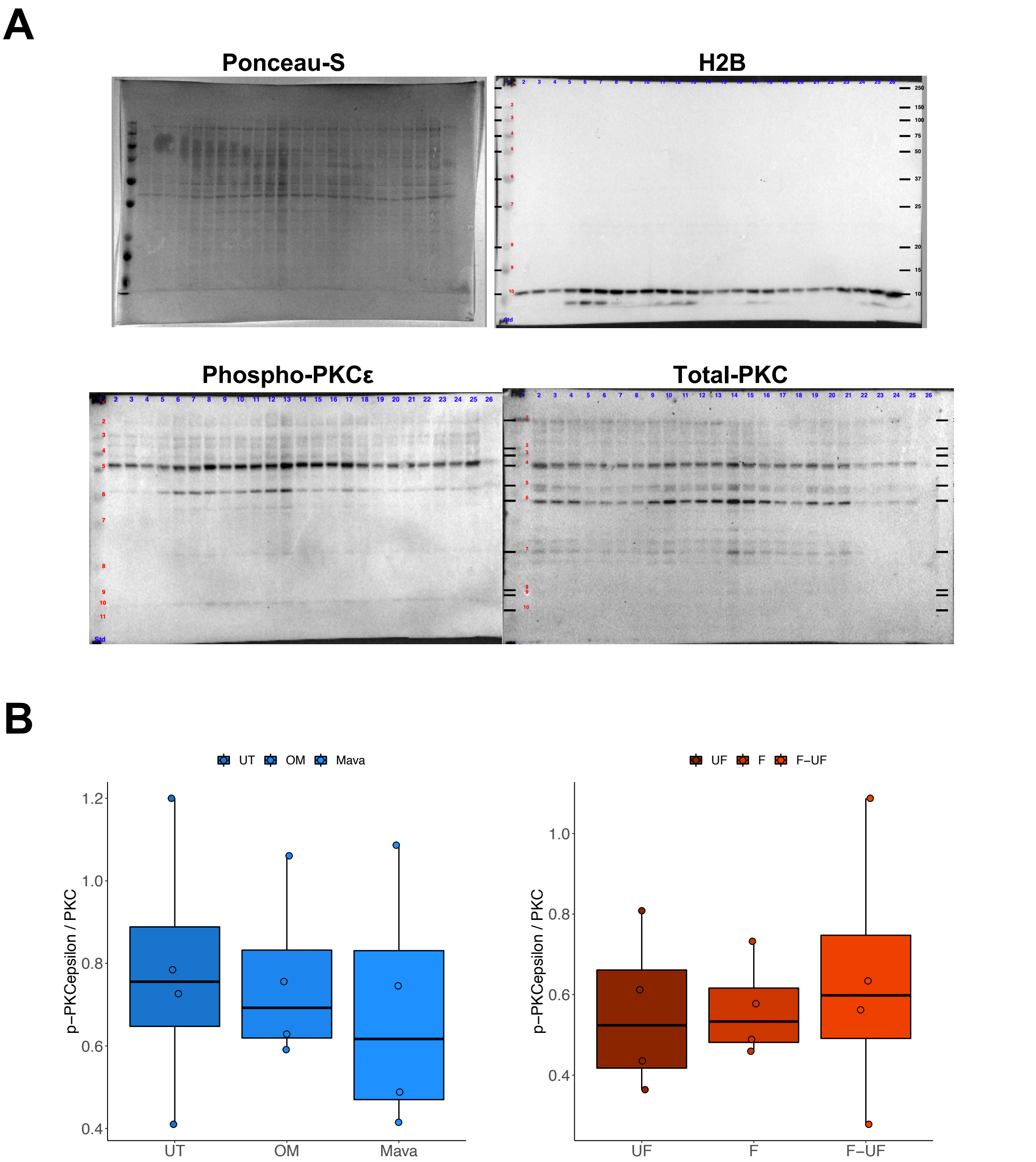

Figure S10. PKC signaling Western blot panel. (A) Whole Ponceau S, H2B, phospho-PKCε, and total PKC Western blots. (B) Quantification of total PKC to H2B for the chemical unloading model composed by untreated (UT), omecamtiv mecarbil (OM), and mavacamten (Mava) (*left*); and the mechanical unloading model composed by unflexed (UF), flexed (F), and flexed-unflexed (F-UF) samples (*right*). Data is presented as mean ± SD (N = 4).

A

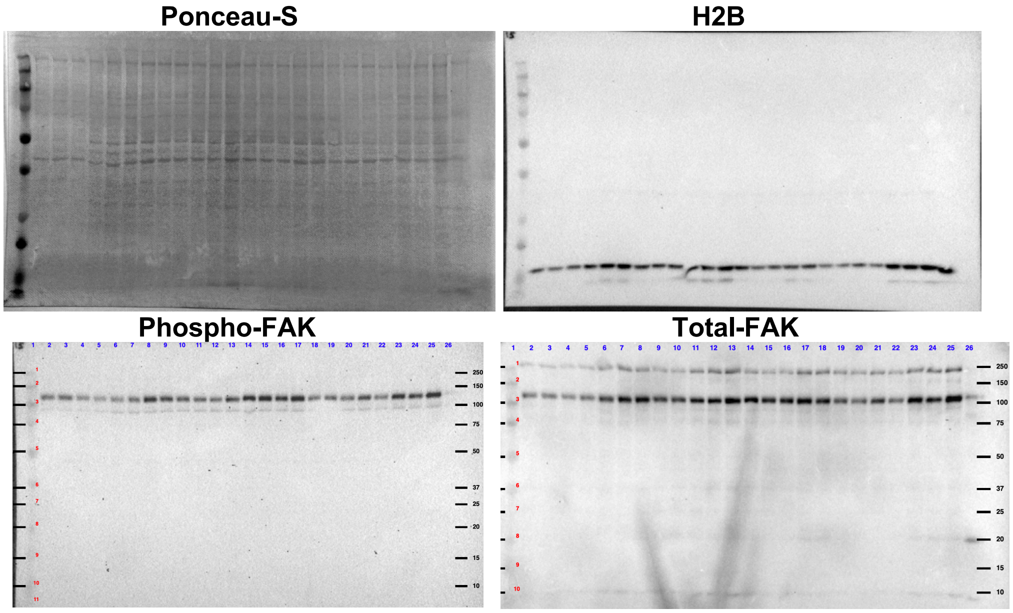

B

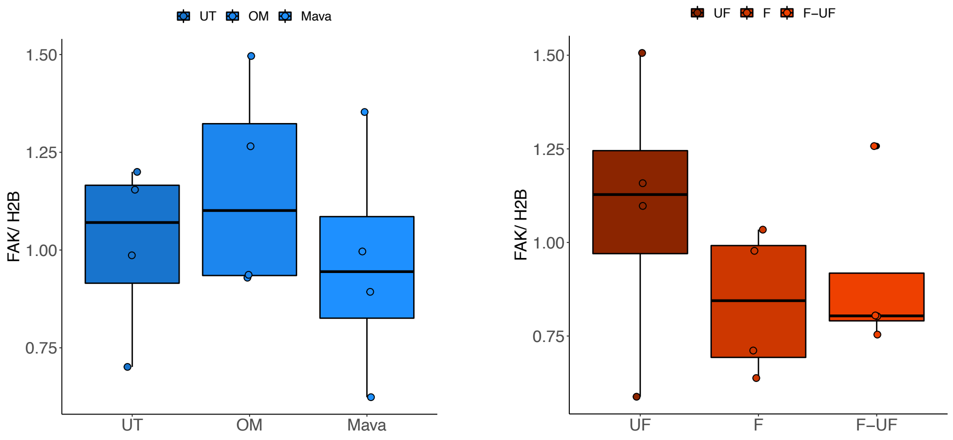

Figure S11. FAK signaling Western blot panel. (A) Whole Ponceau S, H2B, phospho-FAK, and total FAK Western blots. (B) Quantification of total FAK to H2B for the chemical unloading model composed by untreated (UT), omecamtiv mecarbil (OM), and mavacamten (Mava) (*left*); and the mechanical unloading model composed by unflexed (UF), flexed (F), and flexed-unflexed (F-UF) samples (*right*). Data is presented as mean ± SD (N = 4).
